# Supplementary material for: Electrochemically mediated disproportionation for selective formaldehyde upcycling in acid
Source: Nat Commun. 2026 Mar 18;17:4120. doi: 10.1038/s41467-026-70739-y (PMC13150010; doi:10.1038/s41467-026-70739-y)
Supplement: Supplementary file 1 — Supplementary Information [file 41467_2026_70739_MOESM1_ESM.pdf]

Supplementary Information

Electrochemically mediated disproportionation for selective formaldehyde upcycling in acid

Yun Song[1,2]†, Zhaohua Zhu[1]†, Tridip Das[3]†, Aarya D. Riasati[3], Jianjun Su[1,2], Weihua Guo[1,2], Yong Liu[1,2], Geng Li[1,2], Yinger Xin[1,2], Qiang Zhang[1,2], Mingming He[1,2], Ruixuan Wang[1,2], Rui Xue[1,2], Shenlong Zhao[4], Chuan Xia[5], Ben Zhong Tang[6,7], Marc Robert[8], Xin Wang[1], William A. Goddard III[3], Ruquan Ye[1,2,]*

[1]Department of Chemistry and State Key Laboratory of Marine Environmental Health, City University of Hong Kong, Hong Kong 999077, China.

[2]City University of Hong Kong Shenzhen Research Institute, Shenzhen, Guangdong 518057, China.

[3]Materials and Process Simulation Center, California Institute of Technology, Pasadena, CA, USA.

[4]CAS Key Laboratory of Nanosystem and Hierarchical Fabrication, CAS Center for Excellence in Nanoscience, National Center for Nanoscience and Technology, Beijing, P. R. China

[5]School of Materials and Energy, University of Electronic Science and Technology of China, Chengdu, 611731 China

[6]Guangdong Basic Research Center of Excellent for Aggregate Science, School of Science and Engineering, Shenzhen Institute of Molecular Aggregate Science and Engineering, The Chinese University of Hong Kong, Shenzhen (CUHK-Shenzhen), Longgang District, Shenzhen, Guangdong 518172, China.

[7]Department of Chemistry and the Hong Kong Branch of Chinese National Engineering Research Center for Tissue Restoration and Reconstruction, The Hong Kong University of Science and Technology, Hong Kong, China

[8]Sorbonne Université, CNRS, Institut Parisien de Chimie Moléculaire, Institut Universitaire de France (IUF), Paris, F-75005 France.

*Corresponding email. ruquanye@cityu.edu.hk

†These authors contributed equally to this work.

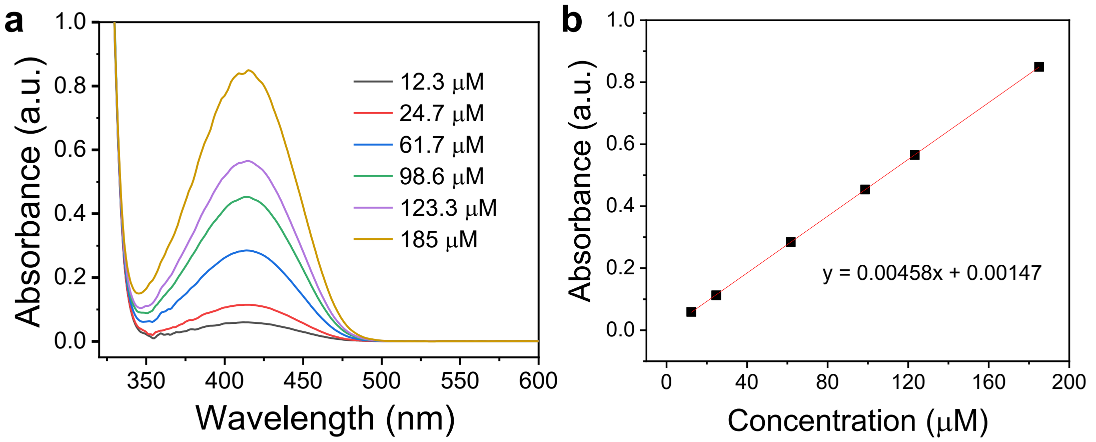

Figure S1. **a** UV-vis absorption spectra of the product from the reaction between acetyl acetone and HCHO of different known concentrations. **b** Calibration curve obtained by plotting the HCHO concentration against absorption peak intensity at λ = 413 nm. Source data for Fig. S1 are provided as a Source Data file.

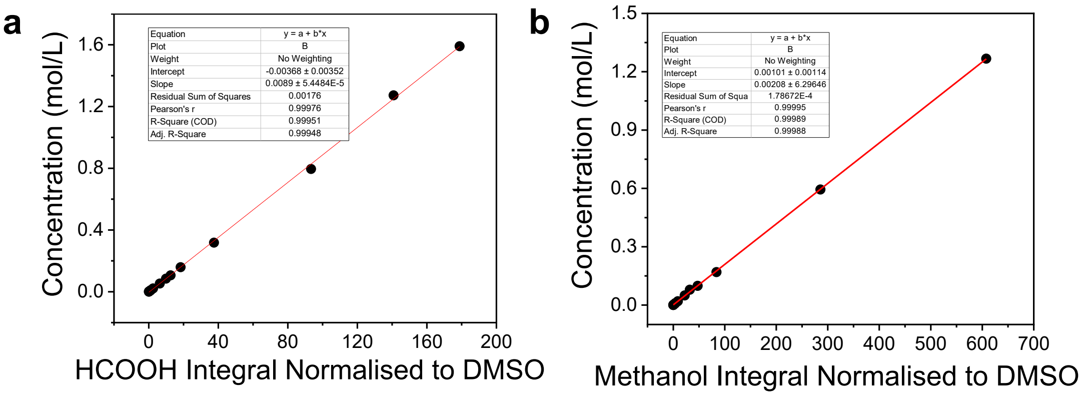

Figure S2. Calibration curves for the ^{1}H NMR quantification of **a** formic acid and **b** methanol using DMSO as an internal standard. Source data for Fig. S2 are provided as a Source Data file.

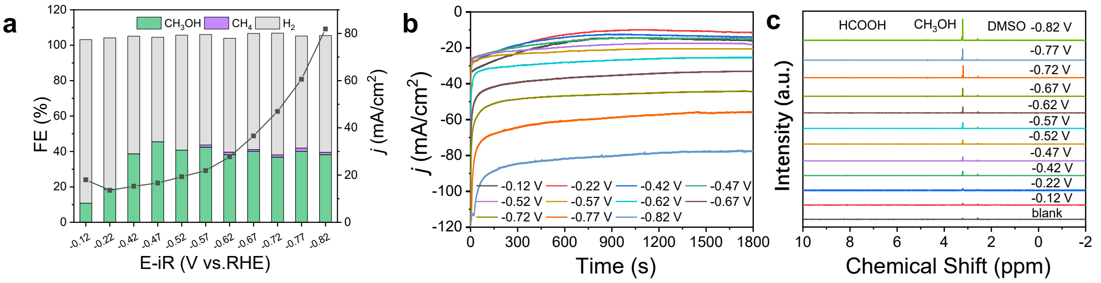

Figure S3. Electrocatalytic FA reduction performance of CuTAPc in an H-cell, catholyte:1 M FA + 0.5 M K_2SO_4 + 0.05 M H_2SO_4, anolyte: 0.5 M H_2SO_4. **a** FE_{CH3OH} and current density, **b** Chronoamperometric curves, and **c** ^{1}H NMR spectra at different potentials. Source data for Fig. S3 are provided as a Source Data file.

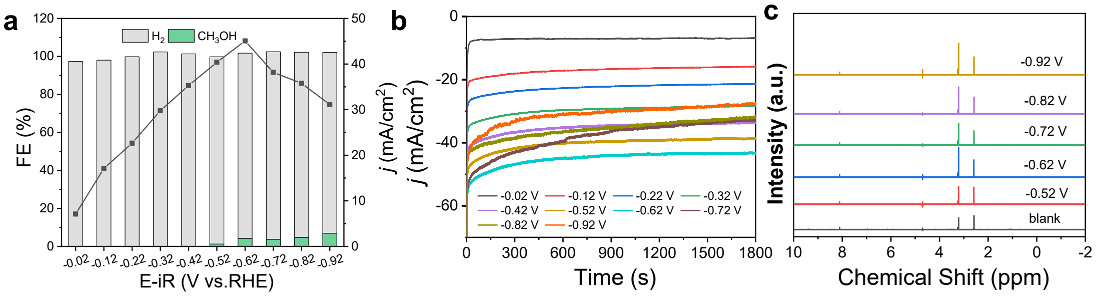

Figure S4. Electrocatalytic FA reduction performance of CoTAPc in an H-cell, catholyte:1 M FA + 0.5 M K_2SO_4 + 0.05 M H_2SO_4, anolyte: 0.5 M H_2SO_4. **a** FE_{CH3OH} and current density, **b** Chronoamperometric curves, and **c** ^{1}H NMR spectra at different potentials. Source data for Fig. S4are provided as a Source Data file.

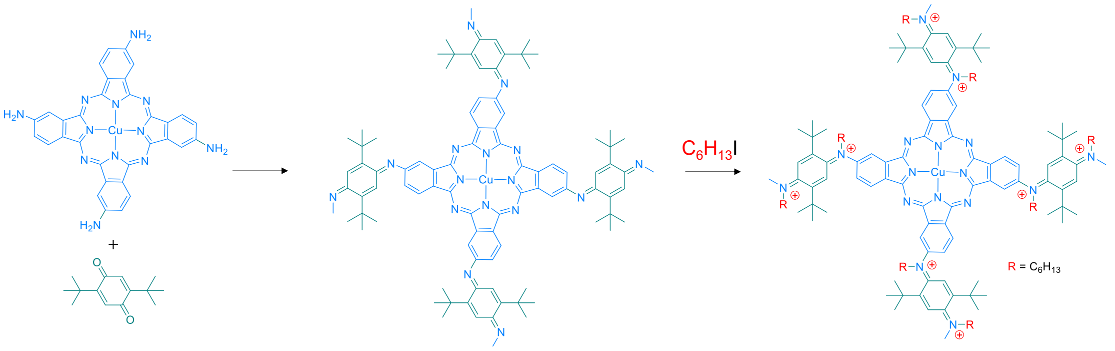

Figure S5. Scheme for the preparation of CuTAPc-layer.

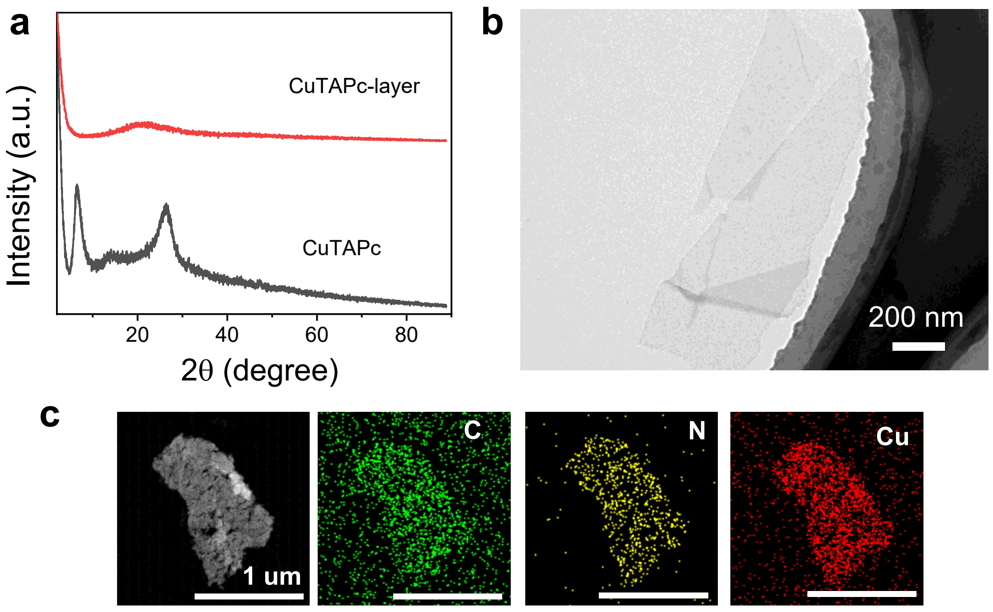

Figure S6. **a** XRD, **b** TEM image and **c** EDS mapping of CuTAPc-layer. Source data for Fig. S6 are provided as a Source Data file.

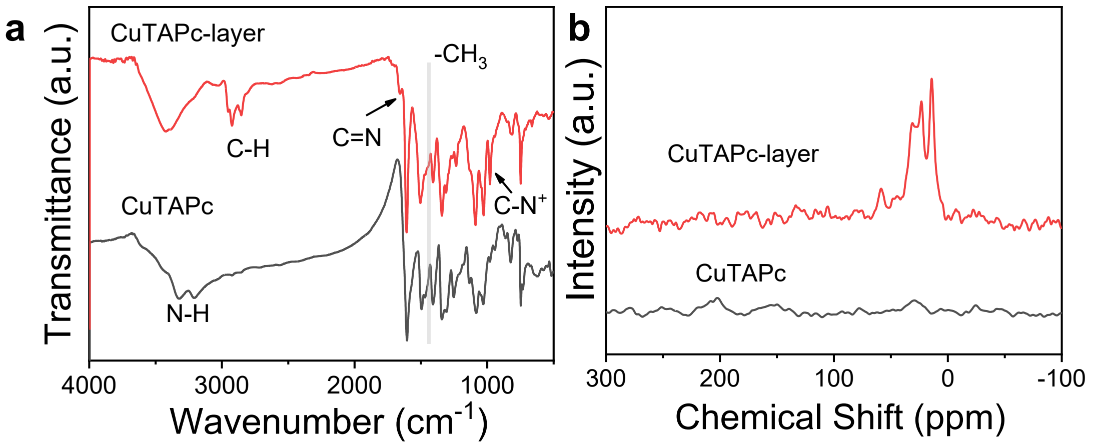

Figure S7. **a** FTIR and **b** ^{13}C NMR spectra of CuTAPc and CuTAPc-layer. Source data for Fig. S7 are provided as a Source Data file.

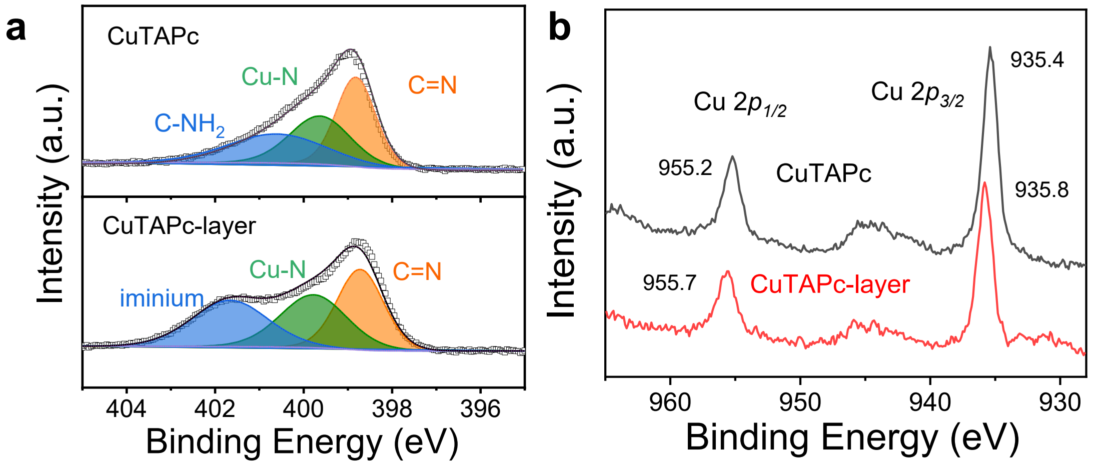

Figure S8. **a** N 1*s* and **b** Cu 2*p* XPS spectra of CuTAPc and CuTAPc-layer. Source data for Fig. S8 are provided as a Source Data file.

For the N 1*s* spectra (Fig. S8a), the peak at 401.7 eV corresponds to iminium N; the deconvolution of N 1*s* spectra suggests that the area ratio of iminium N (bond with alkyl chains), pyrrolic N (Cu-N bond), and pyridinic N (bond with outer carbon of CuPc ring) is 0.92:0.95:1.

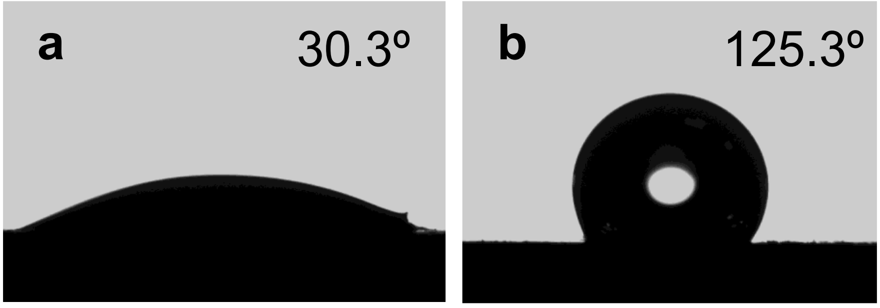

Figure S9. Contact angle measurements of water droplets on **a** CuTAPc and **b** CuTAPc-layer.

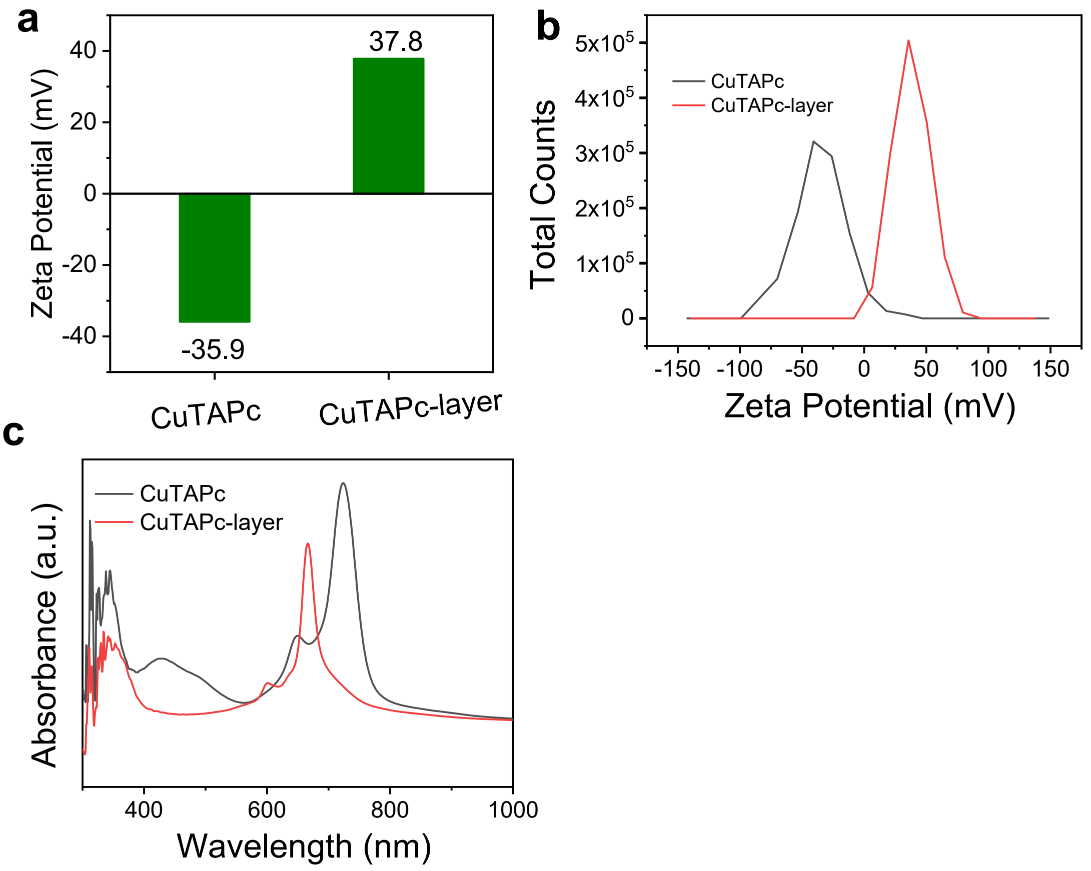

Figure S10. **a** Zeta potential, **b** Zeta potential distribution, and **c** UV-vis spectra of CuTAPc and CuTAPc-layer. Source data for Fig. S10 are provided as a Source Data file.

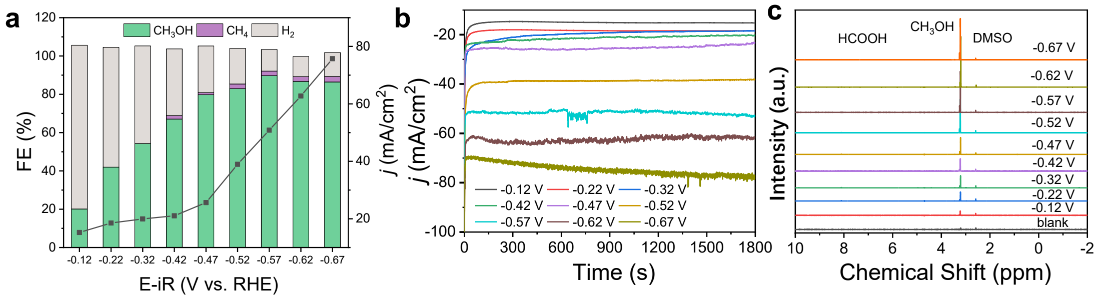

Figure S11. Electrocatalytic FA reduction performance of CuTAPc-layer in an H-cell, catholyte:1 M FA + 0.5 M K_2SO_4 + 0.05 M H_2SO_4, anolyte: 0.5 M H_2SO_4. **a** Product distribution, **b** Chronoamperometric curves, and **c** ^{1}H NMR spectra at different potentials. Source data for Fig. S11 are provided as a Source Data file.

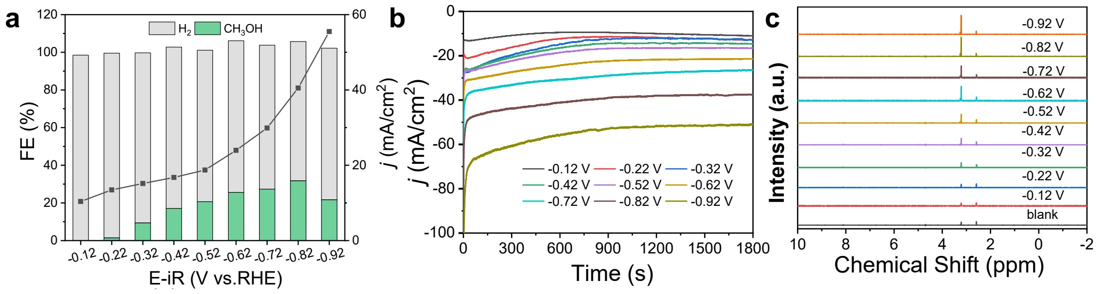

Figure S12. Electrocatalytic FA reduction performance of CoTAPc-layer in an H-cell, catholyte:1 M FA + 0.5 M K_2SO_4 + 0.05 M H_2SO_4, anolyte: 0.5 M H_2SO_4. **a** FE_{CH3OH} and current density, **b** Chronoamperometric curves, and **c** ^1H NMR spectra at different potentials. Source data for Fig. S12 are provided as a Source Data file.

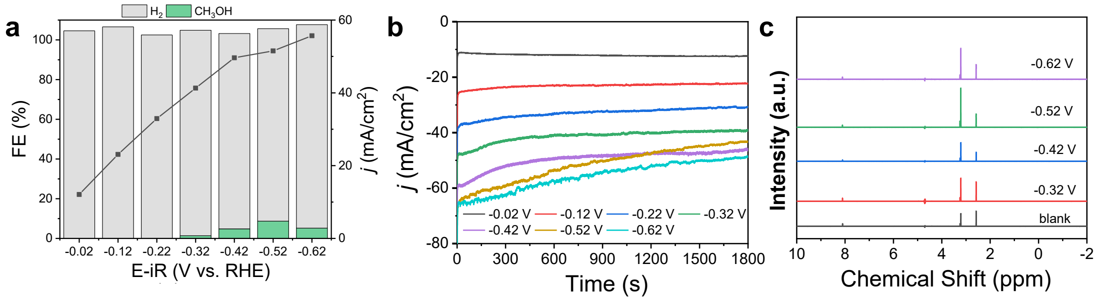

Figure S13. Electrocatalytic FA reduction performance of Cu_2O in an H-cell, catholyte:1 M FA + 0.5 M K_2SO_4 + 0.05 M H_2SO_4, anolyte: 0.5 M H_2SO_4. **a** FE_{CH3OH} and current density, **b** Chronoamperometric curves, and **c** ^1H NMR spectra at different potentials. Source data for Fig. S13 are provided as a Source Data file.

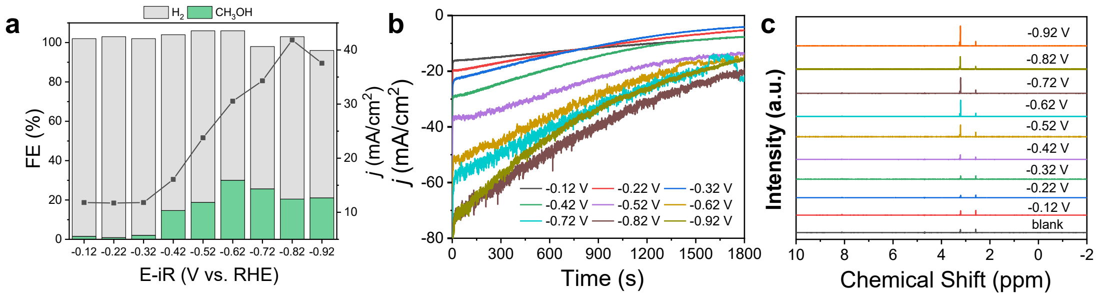

Figure S14. Electrocatalytic FA reduction performance of Cu in an H-cell, catholyte:1 M FA + 0.5 M K_2SO_4 + 0.05 M H_2SO_4, anolyte: 0.5 M H_2SO_4. **a** FE_{CH3OH} and current density, **b** Chronoamperometric curves, and **c** ^1H NMR spectra at different potentials. Source data for Fig. S14 are provided as a Source Data file.

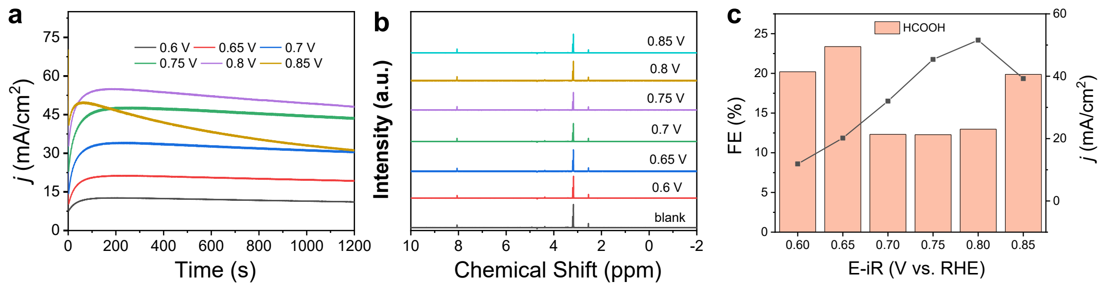

Figure S15. Electrocatalytic FA oxidation performance of 20% Pd/C in an H-cell, anolyte:1 M FA + 0.5 M K_2SO_4 + 0.5 M H_2SO_4, catholyte: 0.5 M H_2SO_4. **a** Chronoamperometric curves and **b** ^{1}H NMR spectra at different potentials. **c** FE_{HCOOH} and current density. Source data for Fig. S15 are provided as a Source Data file.

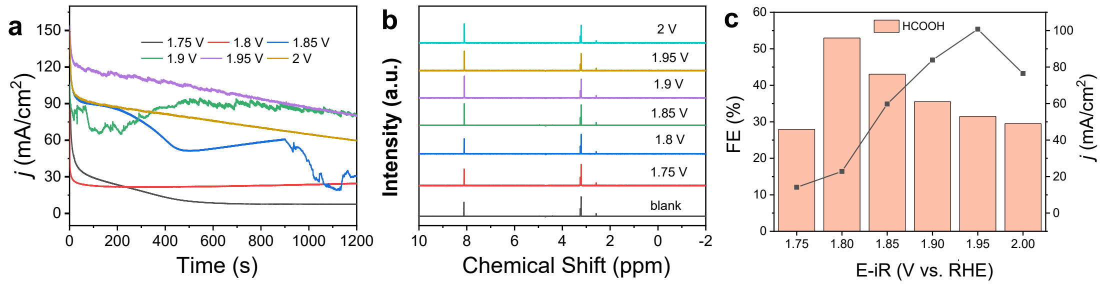

Figure S16. Electrocatalytic FA oxidation performance of 40% Au/C in an H-cell, anolyte:1 M FA + 0.5 M K_2SO_4 + 0.5 M H_2SO_4, catholyte: 0.5 M H_2SO_4. **a** Chronoamperometric curves and **b** ^{1}H NMR spectra at different potentials. **c** FE_{HCOOH} and current density. Source data for Fig. S16 are provided as a Source Data file.

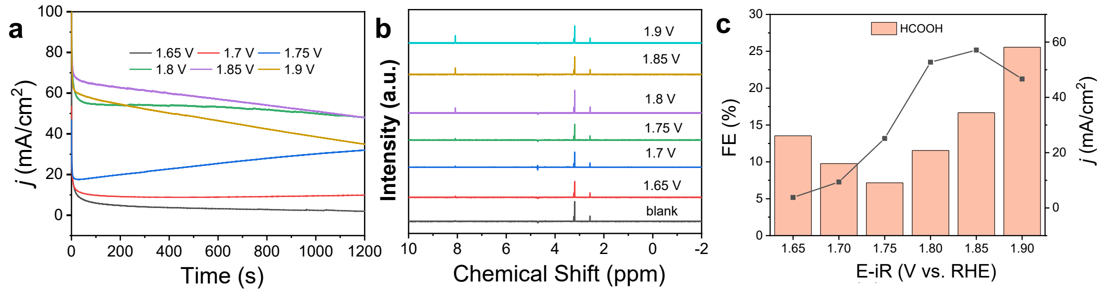

Figure S17. Electrocatalytic FA oxidation performance of Cu_2O in an H-cell, anolyte:1 M FA + 0.5 M K_2SO_4 + 0.5 M H_2SO_4, catholyte: 0.5 M H_2SO_4. **a** Chronoamperometric curves and **b** ^{1}H NMR spectra at different potentials. **c** FE_{HCOOH} and current density. Source data for Fig. S17 are provided as a Source Data file.

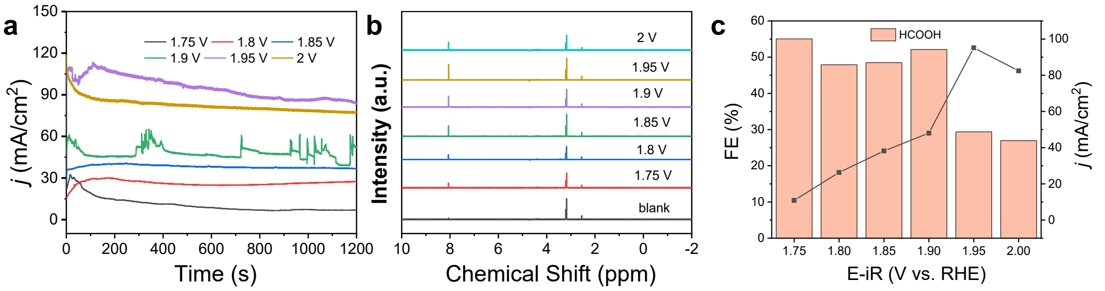

Figure S18. Electrocatalytic FA oxidation performance of Ag in an H-cell, anolyte:1 M FA + 0.5 M K_2SO_4 + 0.5 M H_2SO_4, catholyte: 0.5 M H_2SO_4. **a** Chronoamperometric curves and **b** ^{1}H NMR spectra at different potentials. **c** FE_{HCOOH} and current density. Source data for Fig. S18 are provided as a Source Data file.

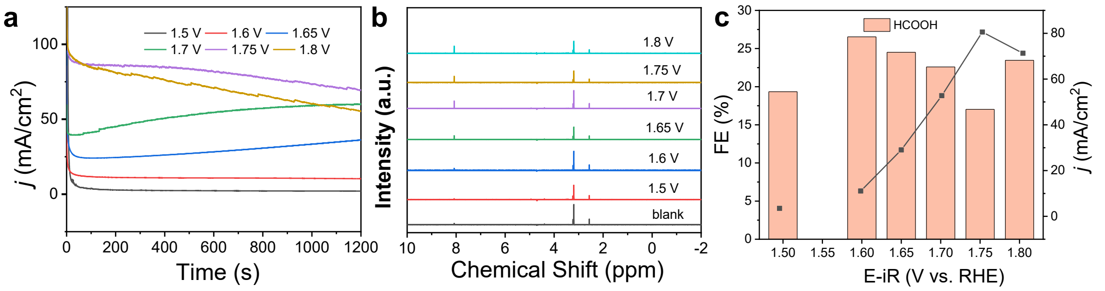

Figure S19. Electrocatalytic FA oxidation performance of Cu in an H-cell, anolyte:1 M FA + 0.5 M K_2SO_4 + 0.5 M H_2SO_4, catholyte: 0.5 M H_2SO_4. **a** Chronoamperometric curves and **b** ^{1}H NMR spectra at different potentials. **c** FE_{HCOOH} and current density. Source data for Fig. S19 are provided as a Source Data file.

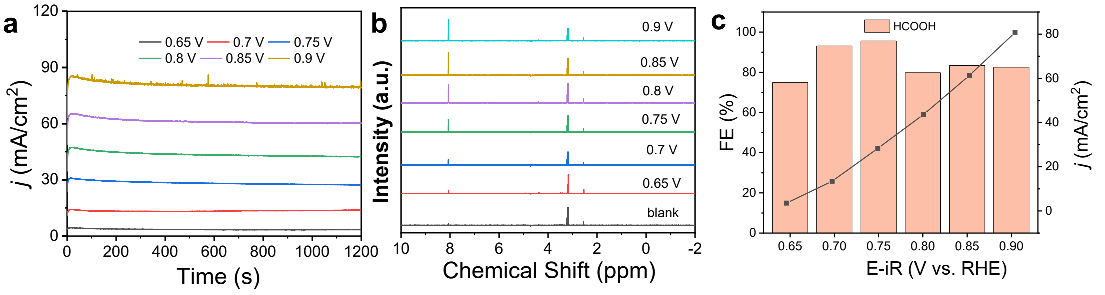

Figure S20. Electrocatalytic FA oxidation performance of Ru in an H-cell, anolyte:1 M FA + 0.5 M K_2SO_4 + 0.5 M H_2SO_4, catholyte: 0.5 M H_2SO_4. **a** Chronoamperometric curves and **b** ^{1}H NMR spectra at different potentials. **c** FE_{HCOOH} and current density. Source data for Fig. S20 are provided as a Source Data file.

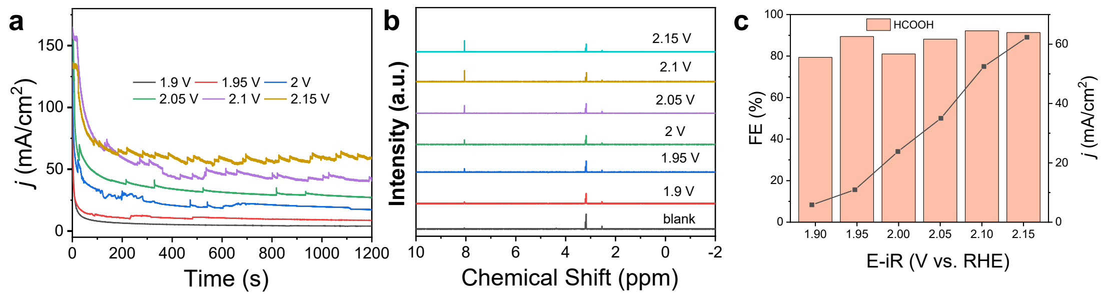

Figure S21. Electrocatalytic FA oxidation performance of Pt in an H-cell, anolyte:1 M FA + 0.5 M K_2SO_4 + 0.5 M H_2SO_4, catholyte: 0.5 M H_2SO_4. **a** Chronoamperometric curves and **b** ^{1}H NMR spectra at different potentials. **c** FE_{HCOOH} and current density. Source data for Fig. S21 are provided as a Source Data file.

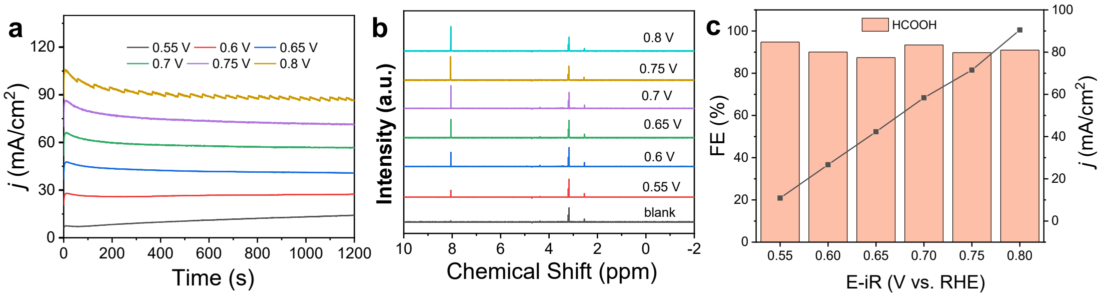

Figure S22. Electrocatalytic FA oxidation performance of Pt_2Ru in an H-cell, anolyte:1 M FA + 0.5 M K_2SO_4 + 0.5 M H_2SO_4, catholyte: 0.5 M H_2SO_4. **a** Chronoamperometric curves and **b** ^{1}H NMR spectra at different potentials. **c** FE_{HCOOH} and current density. Source data for Fig. S22 are provided as a Source Data file.

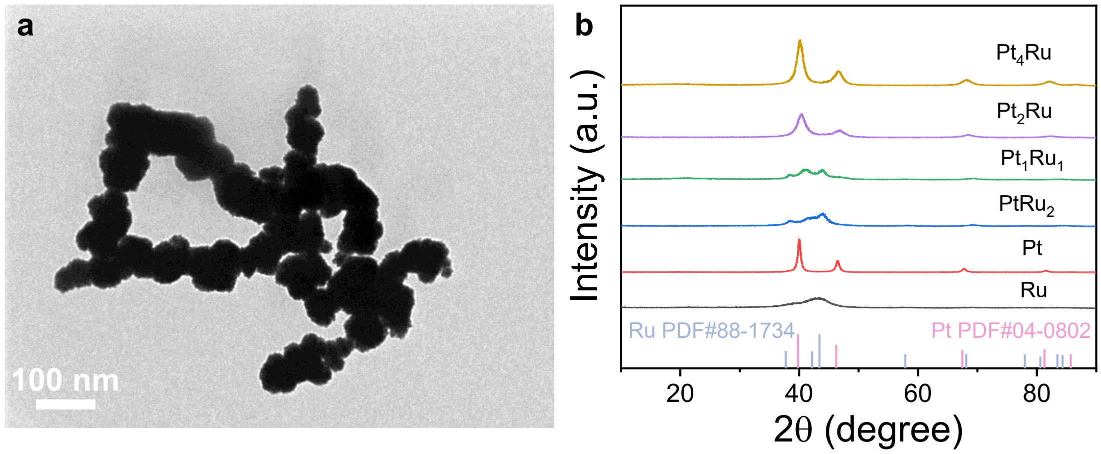

Figure S23. **a** TEM image of Pt_2Ru, **b** XRD patterns of Pt, Ru, $PtRu_2$, Pt_1Ru_1, Pt_2Ru, and Pt_4Ru. Source data for Fig. S23 are provided as a Source Data file.

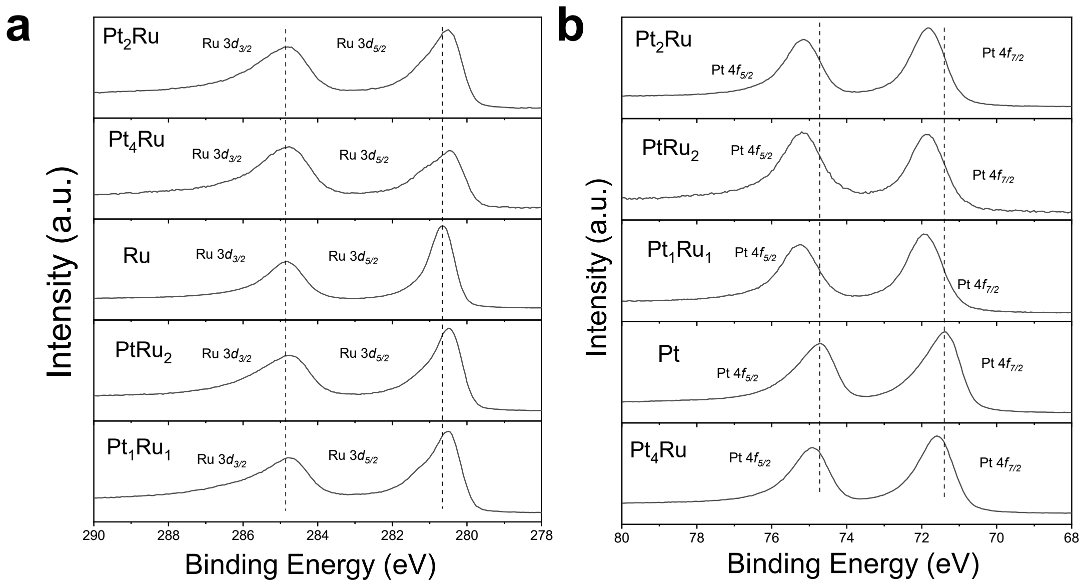

Figure S24. **a** Ru 3*d* and **b** Pt 4*f* XPS spectra of Pt, Ru, $PtRu_2$, Pt_1Ru_1, Pt_2Ru, and Pt_4Ru. Source data for Fig. S24 are provided as a Source Data file.

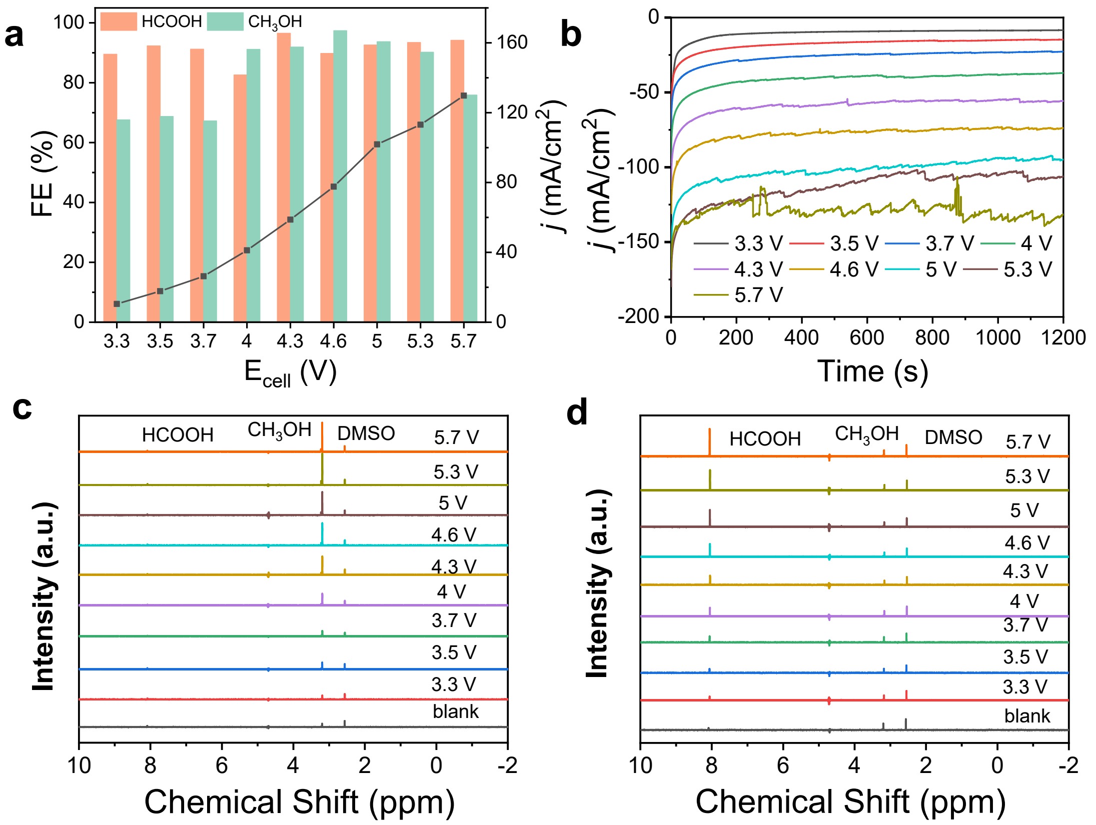

Figure S25. Catalytic performance of the paired CuTAPc-layer and Pt catalysts as the cathode and anode in a combined flow cell, catholyte:1 M FA + 0.5 M K_2SO_4 + 0.05 M H_2SO_4, anolyte: 1 M FA + 0.5 M K_2SO_4 + 0.5 M H_2SO_4. The electrocatalytic performance was measured without iR compensation. **a** Current density and faradic efficiencies, **b** Chronoamperometric curves, **c** ^{1}H NMR spectra of catholytes, and **d** ^{1}H NMR spectra of anolytes at different potentials. Source data for Fig. S25 are provided as a Source Data file.

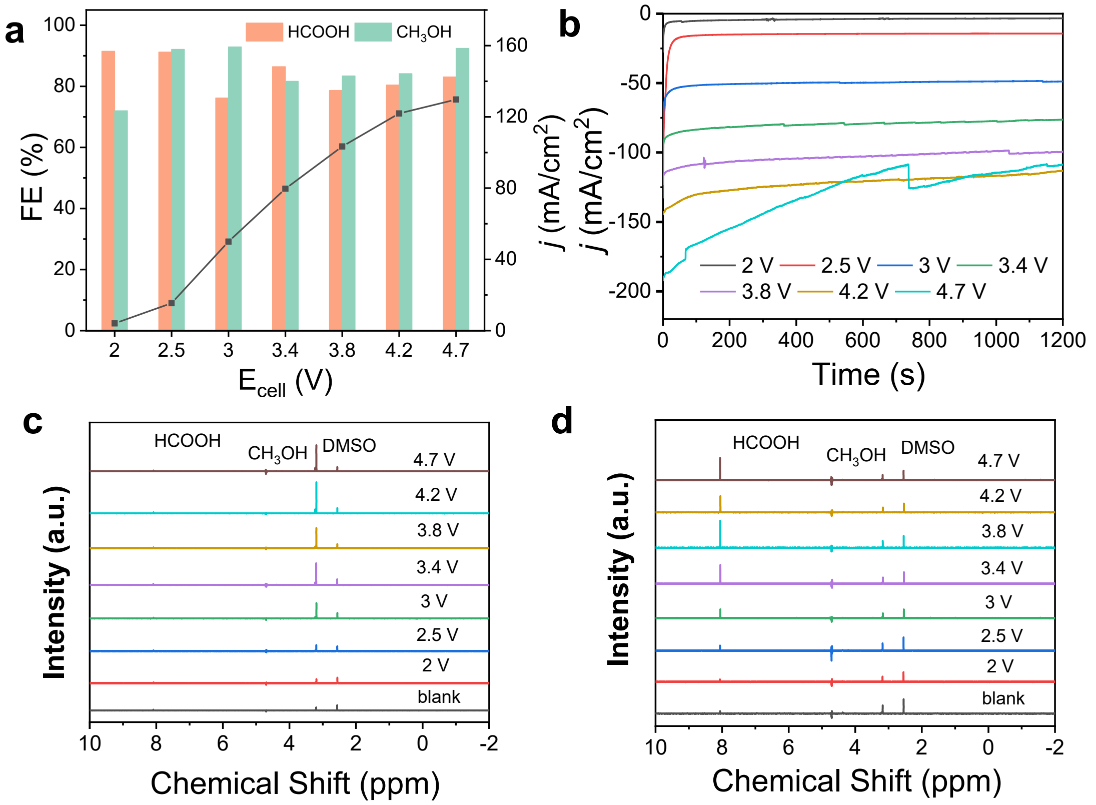

Figure S26. Catalytic performance of the paired CuTAPc-layer and Ru catalysts as the cathode and anode in a combined flow cell, catholyte:1 M FA + 0.5 M K_2SO_4 + 0.05 M H_2SO_4, anolyte: 1 M FA + 0.5 M K_2SO_4 + 0.5 M H_2SO_4. The electrocatalytic performance was measured without iR compensation. **a** Current density and faradic efficiencies, **b** Chronoamperometric curves, **c** ^{1}H NMR spectra of catholytes, and **d** ^{1}H NMR spectra of anolytes at different potentials. Source data for Fig. S26 are provided as a Source Data file.

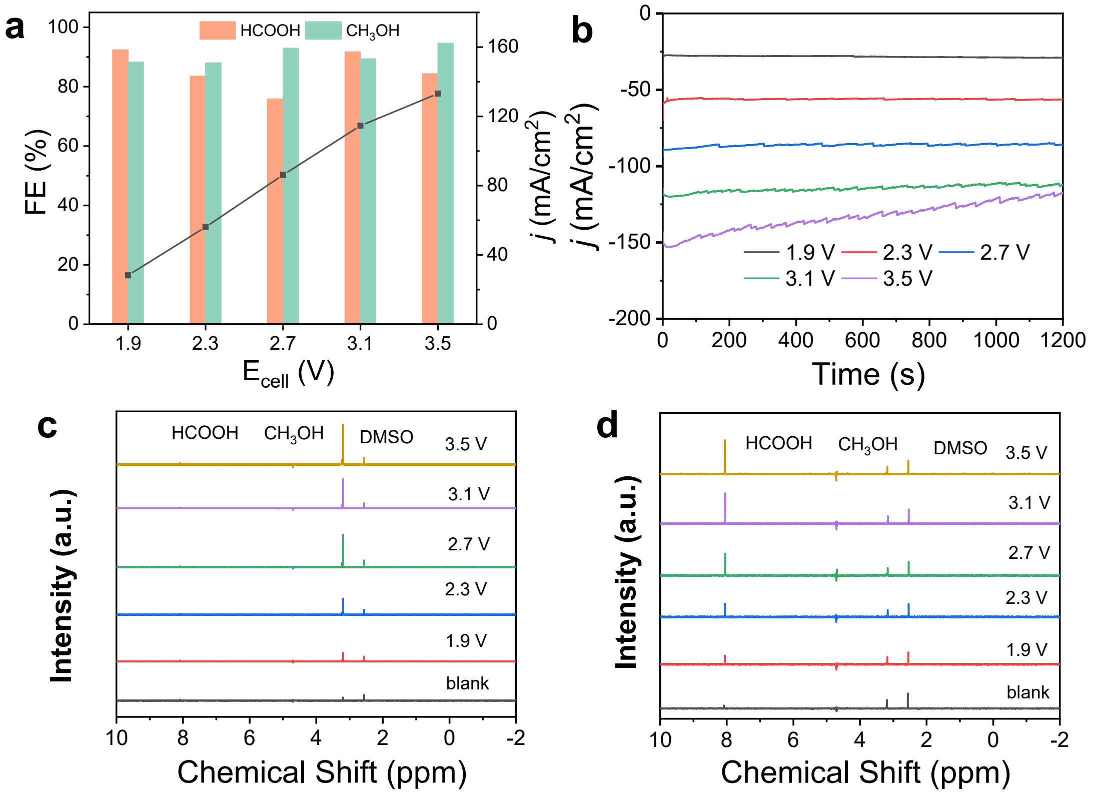

Figure S27. Catalytic performance of the paired CuTAPc-layer and Pt_1Ru_1 catalysts as the cathode and anode in a combined flow cell, catholyte:1 M FA + 0.5 M K_2SO_4 + 0.05 M H_2SO_4, anolyte: 1 M FA + 0.5 M K_2SO_4 + 0.5 M H_2SO_4. The electrocatalytic performance was measured without iR compensation. **a** Current density and faradic efficiencies, **b** Chronoamperometric curves, **c** ^1H NMR spectra of catholytes, and **d** ^1H NMR spectra of anolytes at different potentials. Source data for Fig. S27 are provided as a Source Data file.

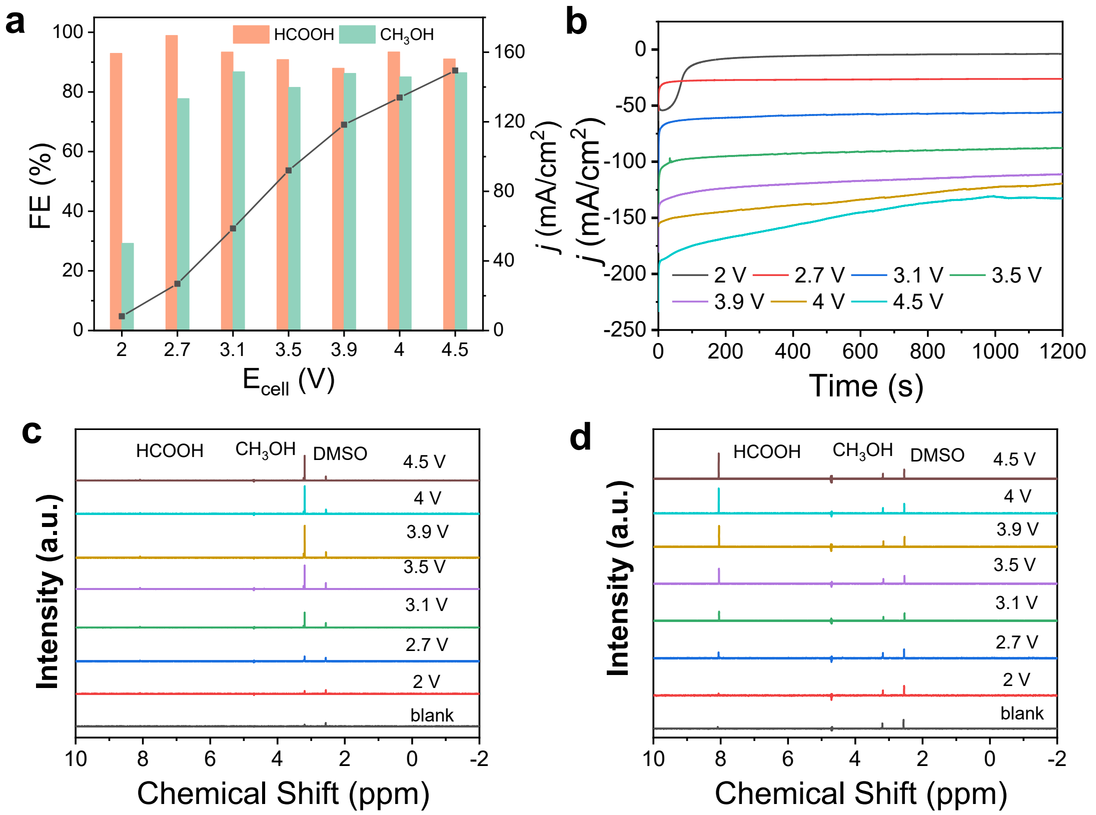

Figure S28. Catalytic performance of the paired CuTAPc-layer and $PtRu_2$ catalysts as the cathode and anode in a combined flow cell, catholyte:1 M FA + 0.5 M K_2SO_4 + 0.05 M H_2SO_4, anolyte: 1 M FA + 0.5 M K_2SO_4 + 0.5 M H_2SO_4. The electrocatalytic performance was measured without iR compensation. **a** Current density and faradic efficiencies, **b** Chronoamperometric curves, **c** ^{1}H NMR spectra of catholytes, and **d** ^{1}H NMR spectra of anolytes at different potentials. Source data for Fig. S28 are provided as a Source Data file.

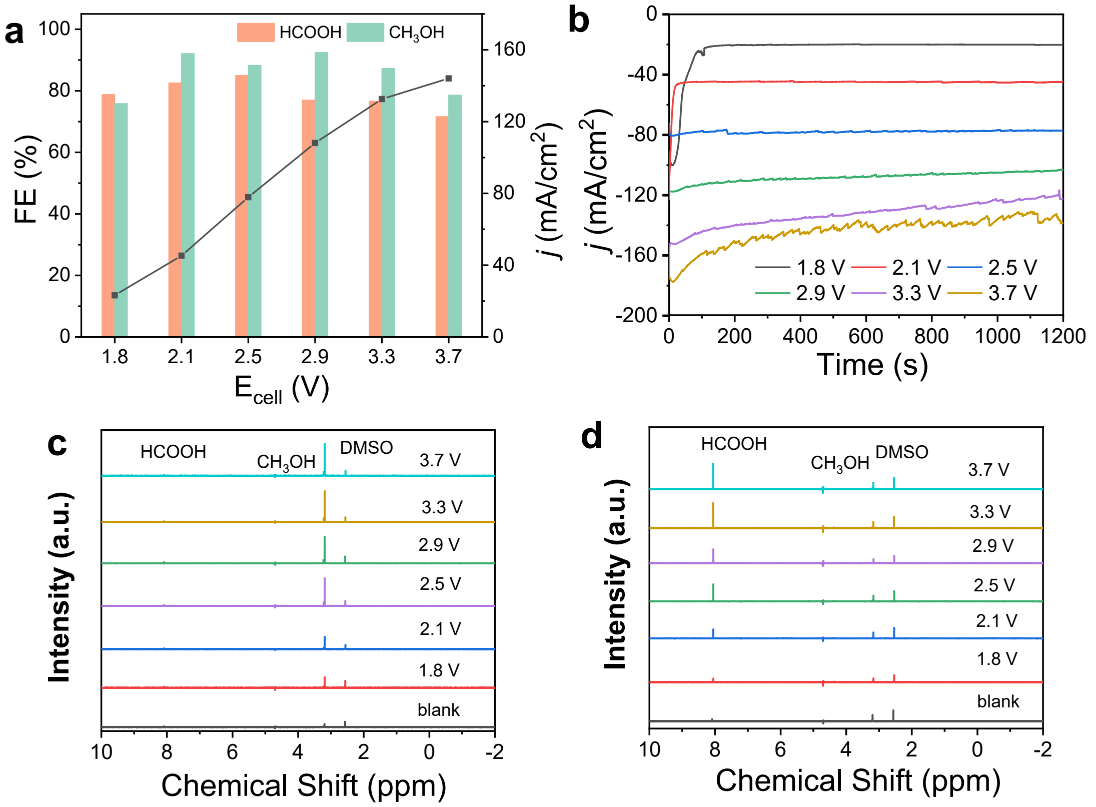

Figure S29. Catalytic performance of the paired CuTAPc-layer and Pt_4Ru catalysts as the cathode and anode in a combined flow cell, catholyte:1 M FA + 0.5 M K_2SO_4 + 0.05 M H_2SO_4, anolyte: 1 M FA + 0.5 M K_2SO_4 + 0.5 M H_2SO_4. The electrocatalytic performance was measured without iR compensation. **a** Current density and faradic efficiencies, **b** Chronoamperometric curves, **c** ^{1}H NMR spectra of catholytes, and **d** ^{1}H NMR spectra of anolytes at different potentials. Source data for Fig. S29 are provided as a Source Data file.

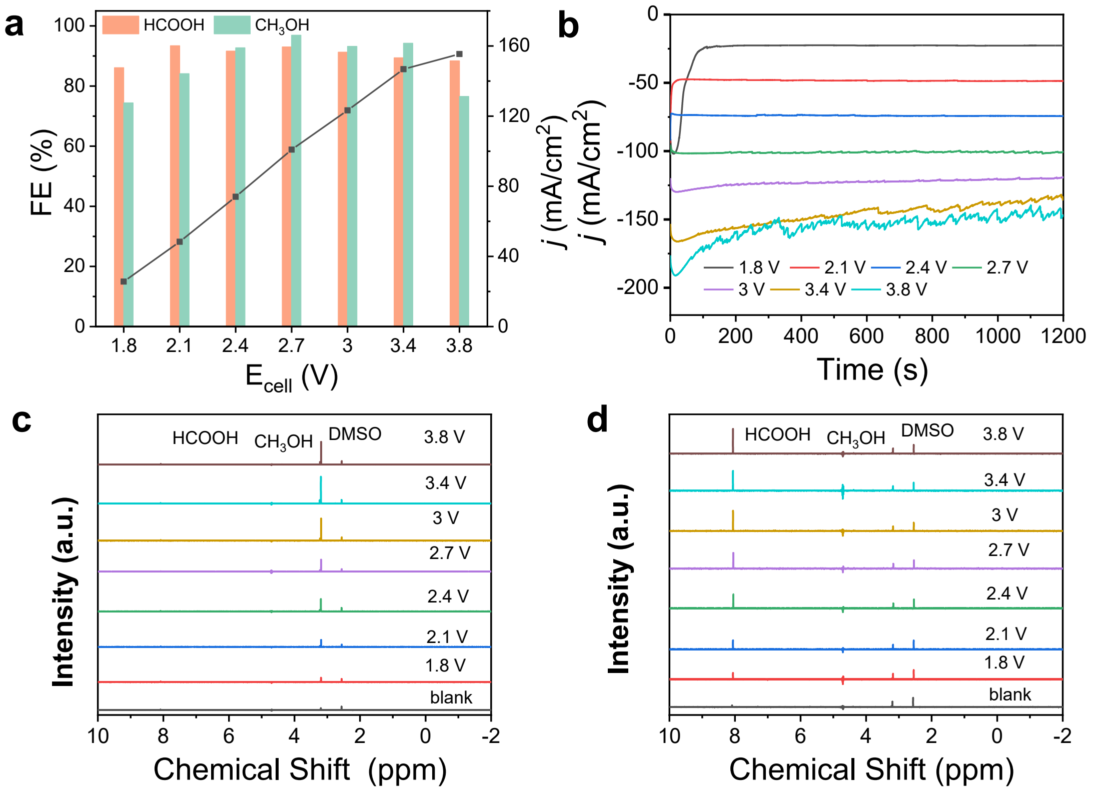

Figure S30. Catalytic performance of the paired CuTAPc-layer and Pt_2Ru catalysts as the cathode and anode in a combined flow cell, catholyte:1 M FA + 0.5 M K_2SO_4 + 0.05 M H_2SO_4, anolyte: 1 M FA + 0.5 M K_2SO_4 + 0.5 M H_2SO_4. The electrocatalytic performance was measured without iR compensation. **a** Current density and faradic efficiencies, **b** Chronoamperometric curves, **c** ^1H NMR spectra of catholytes, and **d** ^1H NMR spectra of anolytes at different potentials. Source data for Fig. S30 are provided as a Source Data file.

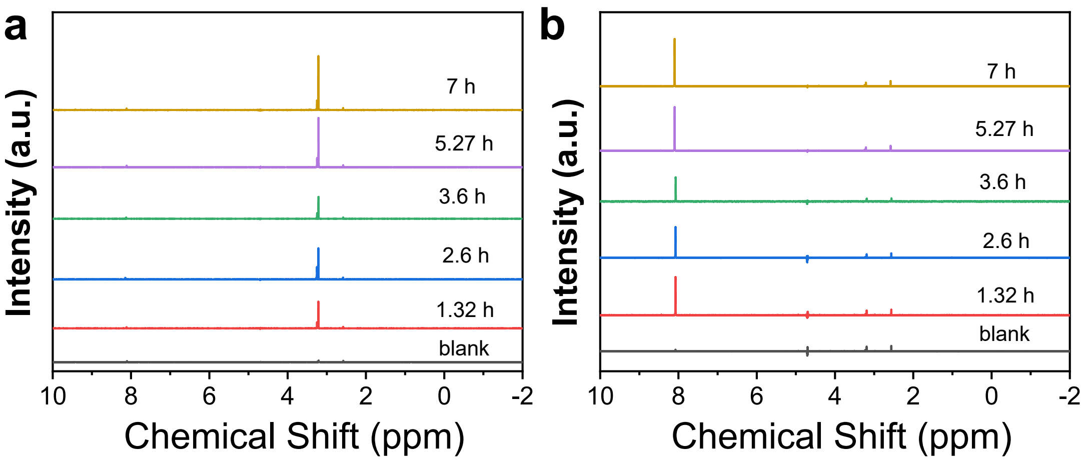

Figure S31. Stability test. **a,b** ^1H NMR spectra of catholytes and anolytes at different potentials. Source data for Fig. S31 are provided as a Source Data file.

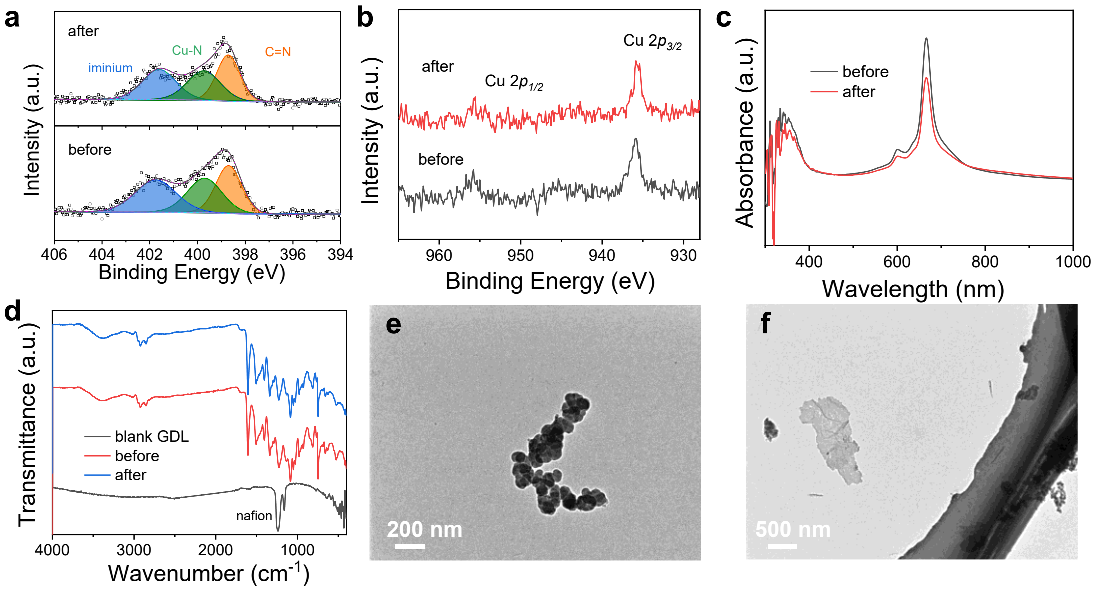

Figure S32. Characterizations of CuTAPc-layer before and after stability tests. **a** N 1*s* XPS spectra, **b** Cu 2*p* XPS spectra, **c** UV-vis spectra, **d** FTIR spectra, **e** TEM image of carbon black, and **f** TEM image of CuTAPc-layer at a full cell voltage of 3 V, catholyte:1 M FA + 0.5 M K_2SO_4 + 0.05 M H_2SO_4. Source data for Fig. S32 are provided as a Source Data file.

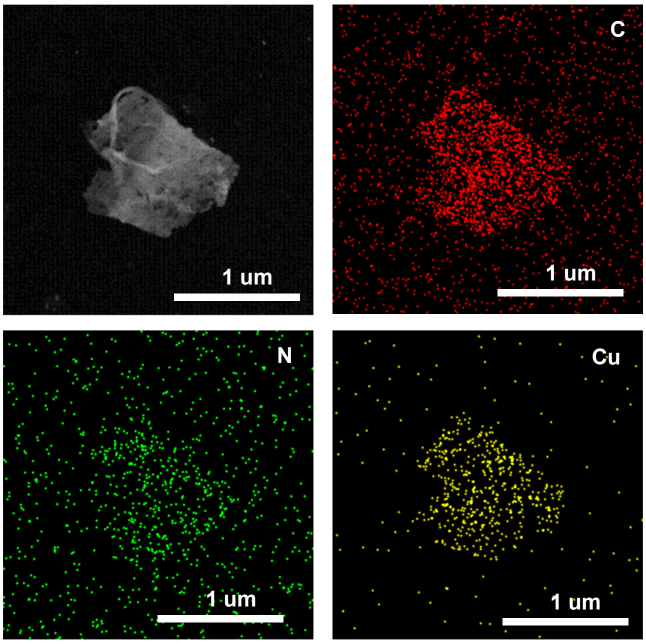

Figure S33. EDS mapping of CuTAPc-layer after stability tests.

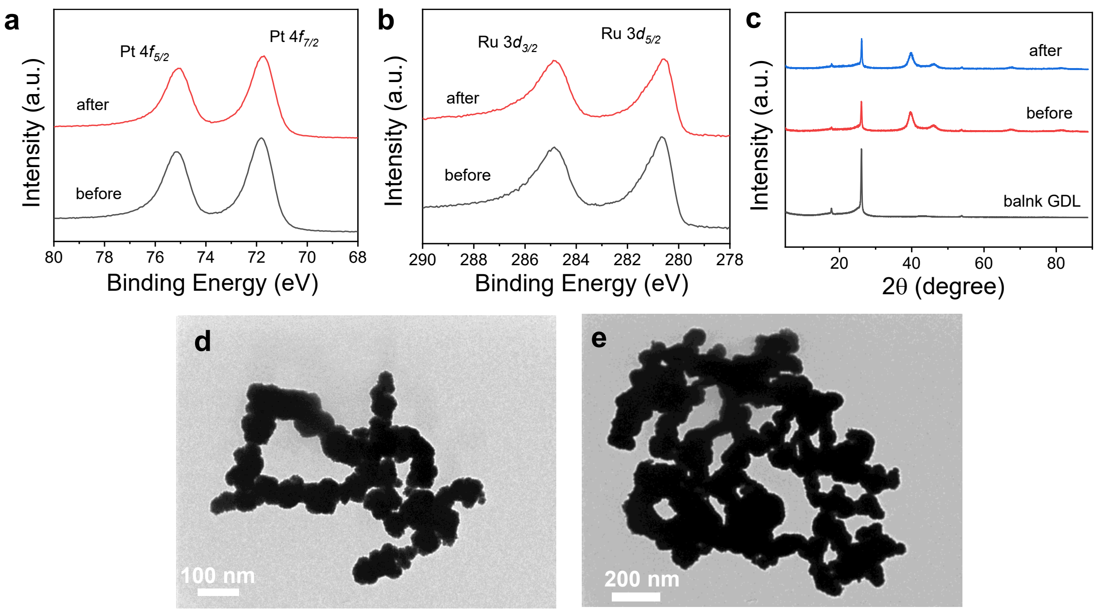

Figure S34. Characterizations of Pt_2Ru before and after stability tests. **a** Pt 4*f* XPS spectra, **b** Ru 3*d* XPS spectra, and **c** XRD pattern of Pt_2Ru at a full cell voltage of 3 V, anolyte: 1 M FA + 0.5 M K_2SO_4 + 0.5 M H_2SO_4. **d,e** TEM images of Pt_2Ru before and after electrolysis. Source data for Fig. S34 are provided as a Source Data file.

The concentration of copper in the catholyte is determined using ICP-OES, and the content of copper is found to be below the detection limit. The structure of CuTAPc-layer after stability test is characterized. As shown in Fig. S32f and S33, CuTAPc-layer maintains its nanosheet morphology with copper element homogenously dispersed. XPS spectra (Fig. S32a,b) demonstrate that both the N 1*s* and Cu 2*p* spectra remain virtually unchanged before and after testing, indicating that the chemical states are well preserved. The chemical structure of CuTAPc-layer after long-time operation is further confirmed by UV-vis and FTIR spectra: the Q band of CuTAPc-layer presents no significant shift, and the retention of iminium band in FTIR spectra collectively confirm the structural stability of the catalyst under operating conditions.

Similarly, Pt_2Ru after long-time operation is also characterized. XPS spectra (Fig. S34) show that the chemical states of Pt and Ru remain unchanged, with an atomic ratio of 1.95:1. XRD analysis confirms that Pt_2Ru retains its crystallographic structure without the formation of new phases. Additionally, TEM images demonstrate that Pt_2Ru preserves spheric shape morphology after long-term testing.

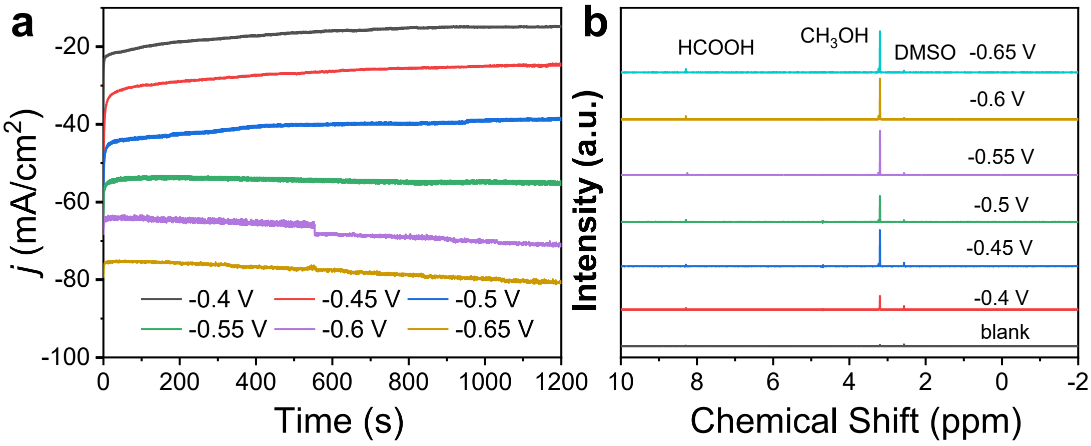

Figure S35. Electrocatalytic FA reduction performance of CuTAPc-layer in an H-cell, catholyte: 0.5 M K_2SO_4 + 1 M FA, anolyte: 0.5 M K_2SO_4. **a** Chronoamperometric curves and **b** ^{1}H NMR spectra at different potentials. Source data for Fig. S35 are provided as a Source Data file.

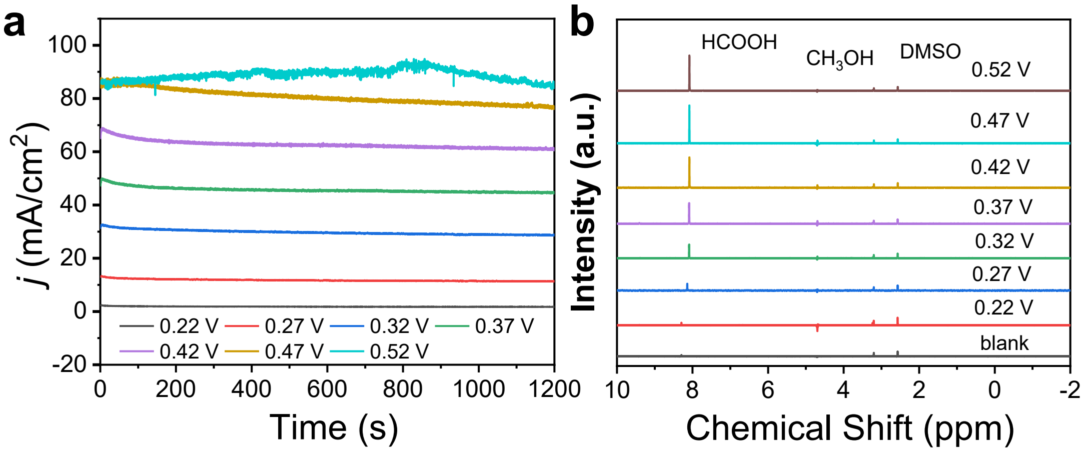

Figure S36. Electrocatalytic FA oxidation performance of Pt_2Ru in an H-cell, anolyte: 0.5 M K_2SO_4 + 1 M FA, catholyte: 0.5 M K_2SO_4. **a** Chronoamperometric curves and **b** ^{1}H NMR spectra at different potentials. Source data for Fig. S36 are provided as a Source Data file.

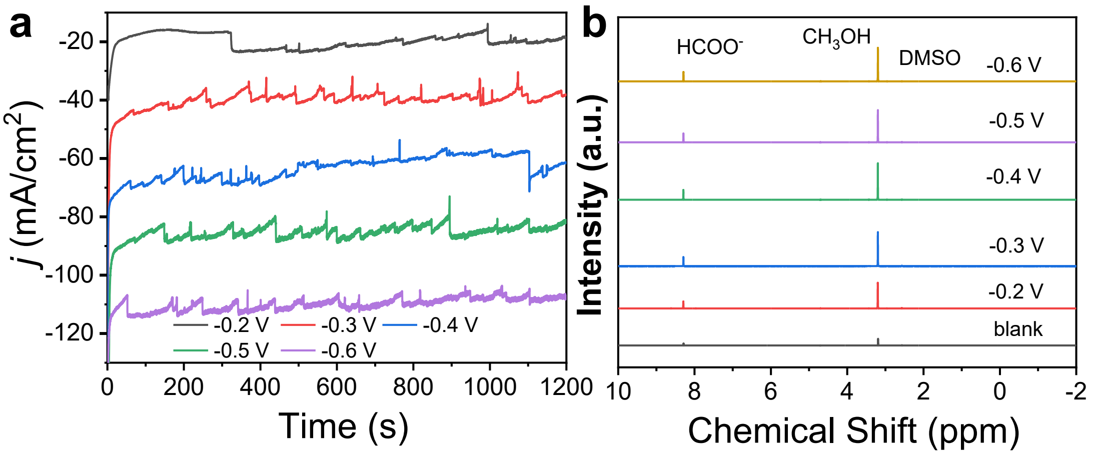

Figure S37. Electrocatalytic FA reduction performance of CuTAPc-layer in an H-cell, catholyte: 1 M KOH + 1 M FA, anolyte: 1 M KOH. **a** Chronoamperometric curves and **b** ^{1}H NMR spectra at different potentials. Source data for Fig. S37 are provided as a Source Data file.

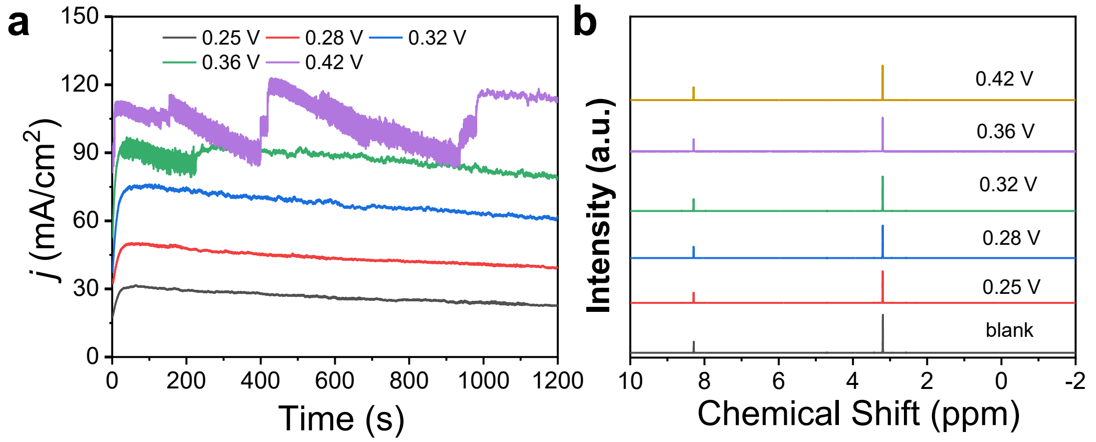

Figure S38. Electrocatalytic FA oxidation performance of Pt_2Ru in an H-cell, anolyte: 1 M KOH + 1 M FA, catholyte: 1 M KOH. **a** Chronoamperometric curves and **b** ^{1}H NMR spectra at different potentials. Source data for Fig. S38 are provided as a Source Data file.

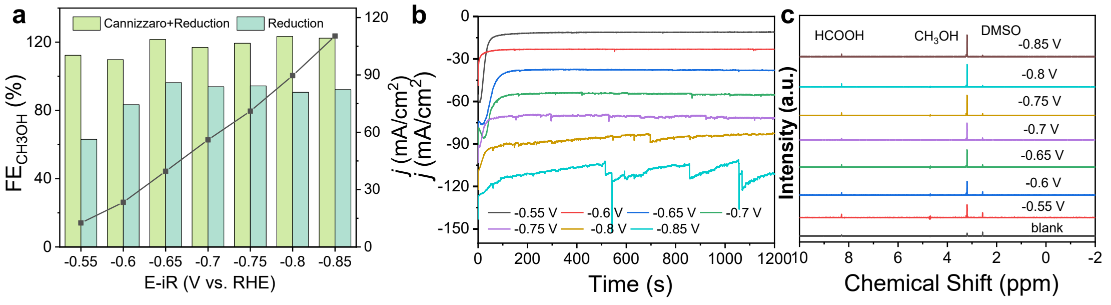

Figure S39. Electrocatalytic FA reduction performance of CuTAPc-layer in a flow cell, catholyte: 0.5 M K_2SO_4 + 1 M FA, anolyte: 0.5 M K_2SO_4. **a** FE_{CH3OH} and current density, **b**

Chronoamperometric curves, and **c** ^{1}H NMR spectra at different potentials. Source data for Fig. S39 are provided as a Source Data file.

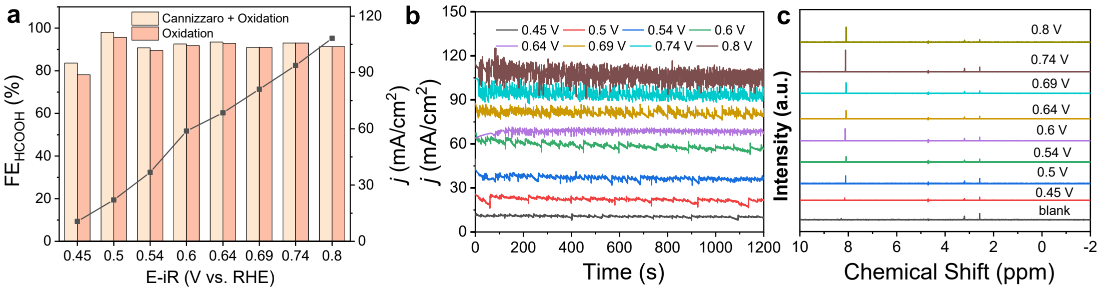

Figure S40. Electrocatalytic FA oxidation performance of Pt_2Ru in a flow cell, anolyte: 0.5 M K_2SO_4 + 1 M FA, catholyte: 0.5 M K_2SO_4. **a** FE_{HCOOH} and current density, **b** Chronoamperometric curves, and **c** ^{1}H NMR spectra at different potentials. Source data for Fig. S40 are provided as a Source Data file.

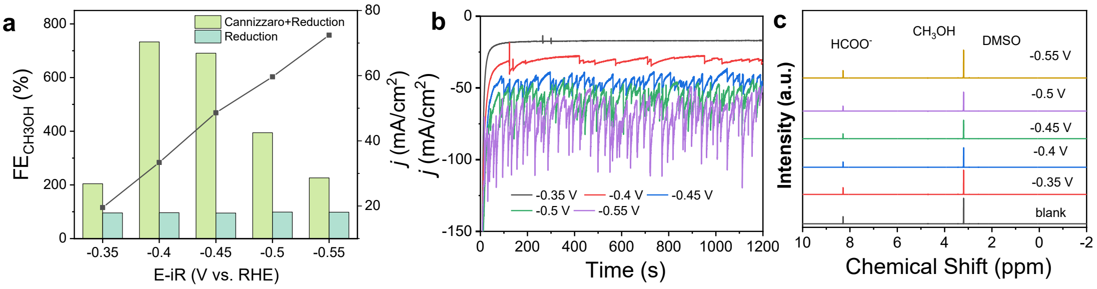

Figure S41. Electrocatalytic FA reduction performance of CuTAPc-layer in a flow cell, catholyte: 1 M FA + 1 M KOH, anolyte: 1 M KOH. **a** FE_{CH3OH} and current density, **b** Chronoamperometric curves, and **c** ^{1}H NMR spectra at different potentials. Source data for Fig. S41 are provided as a Source Data file.

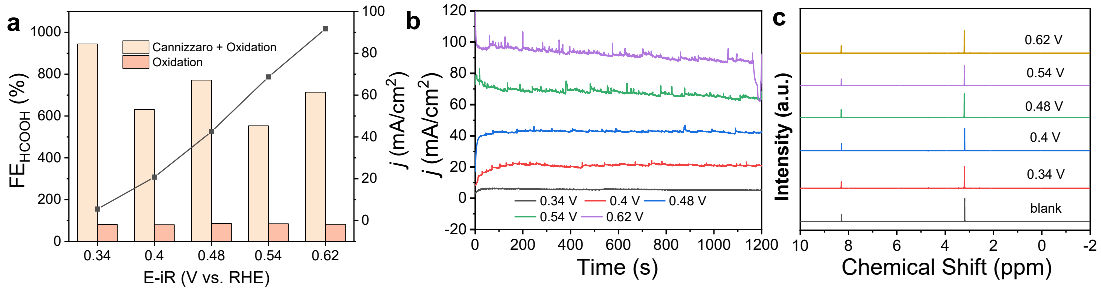

Figure S42. Electrocatalytic FA oxidation performance of Pt_2Ru in a flow cell, anolyte: 1 M KOH + 1 M FA, catholyte: 1 M KOH. **a** FE_{HCOOH} and current density, **b** Chronoamperometric curves, and **c** ^{1}H NMR spectra at different potentials. Source data for Fig. S42 are provided as a Source Data file.

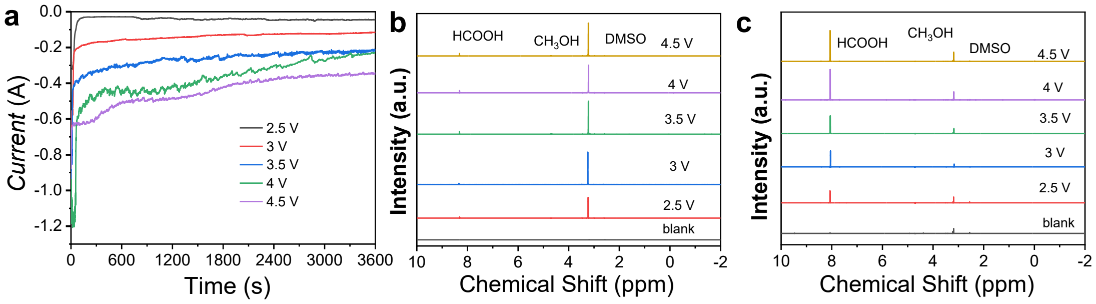

Figure S43. Electrocatalytic conversion of FA in a 4 cm^2 flow cell. **a**, Chronoamperometric curves, **b** ^{1}H NMR spectra of catholytes, and **c** ^{1}H NMR spectra of anolytes at different cell voltages. Source data for Fig. S43 are provided as a Source Data file.

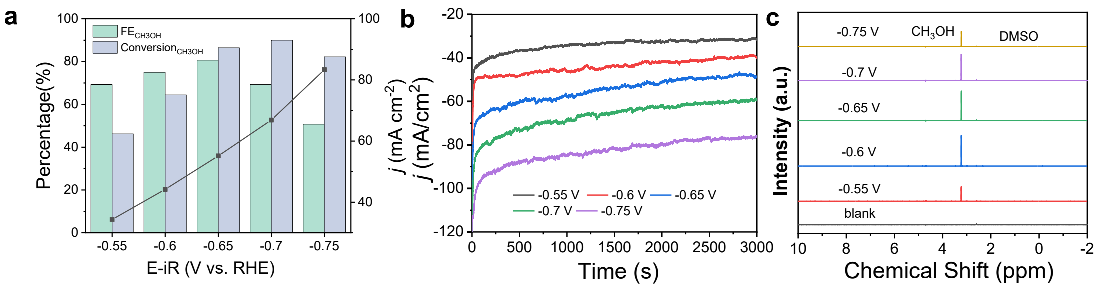

Figure S44. Electrocatalytic FA reduction performance of CuTAPc-layer in an H-cell, catholyte: 0.1 M FA + 0.5 M K_2SO_4 + 0.05 M H_2SO_4, anolyte: 0.5 M H_2SO_4. **a** SPCE$_{CH3OH}$, FE$_{CH3OH}$, and current densities, **b** Chronoamperometric curves, and **c** ^{1}H NMR spectra at different potentials. Source data for Fig. S44 are provided as a Source Data file.

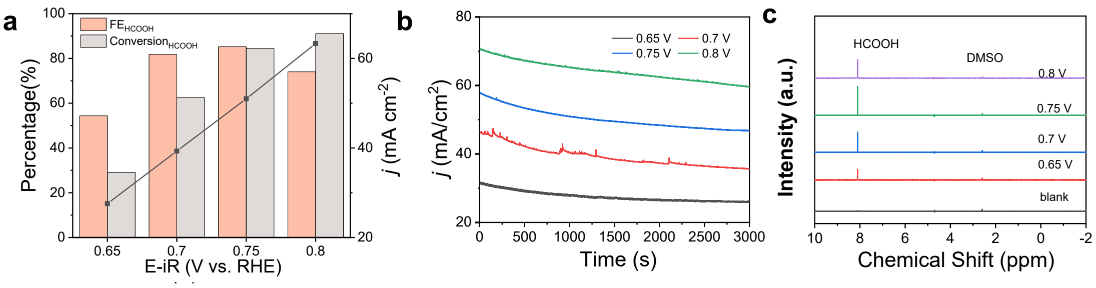

Figure S45. Electrocatalytic FA oxidation performance of Pt_2Ru in an H-cell, anolyte: 0.1 M FA + 0.5 M K_2SO_4 + 0.5 M H_2SO_4, catholyte: 0.5 M H_2SO_4. **a** SPCE$_{HCOOH}$, FE$_{HCOOH}$, and current densities, **b** Chronoamperometric curves, and **c** ^{1}H NMR spectra at different potentials. Source data for Fig. S45 are provided as a Source Data file.

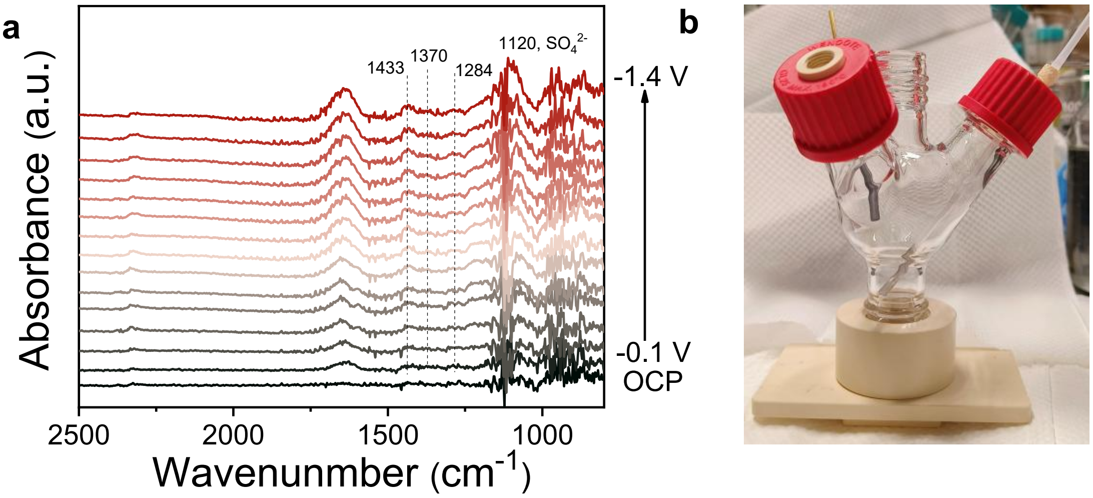

Figure S46. **a** In situ ATR-SEIRAS spectra of electrocatalytic reduction process catalyzed by CuTAPc in 1 M FA + 1 M K_2SO_4 + 0.05 M H_2SO_4. **b** Photograph of the in situ ATR-SEIRAS electrocatalytic cell. Source data for Fig. S46 are provided as a Source Data file.

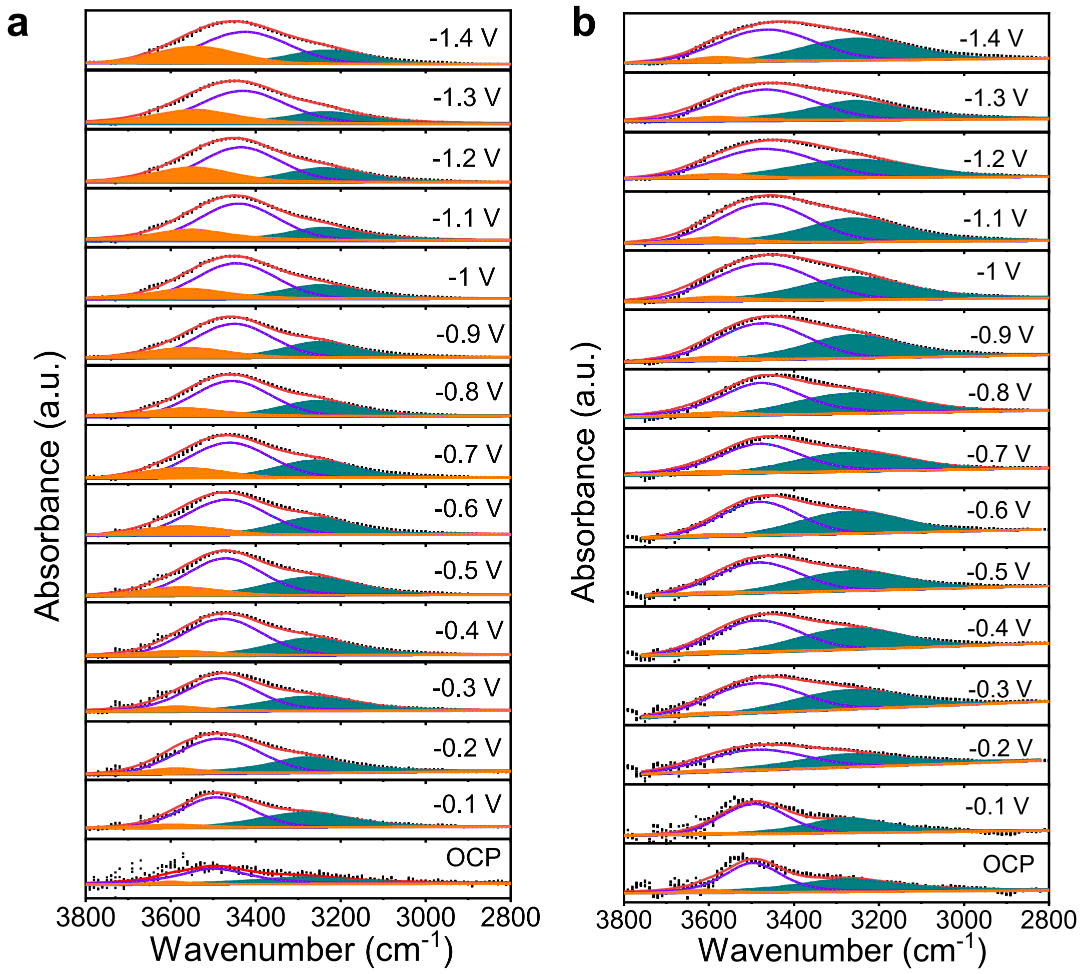

Figure S47. In situ ATR-SEIRAS spectra of interfacial water at varying potentials for **a** CuTAPc and **b** CuTAPc-layer in 1M FA+ 0.5 M K_2SO_4 + 0.05 M H_2SO_4. Source data for Fig. S47 are provided as a Source Data file.

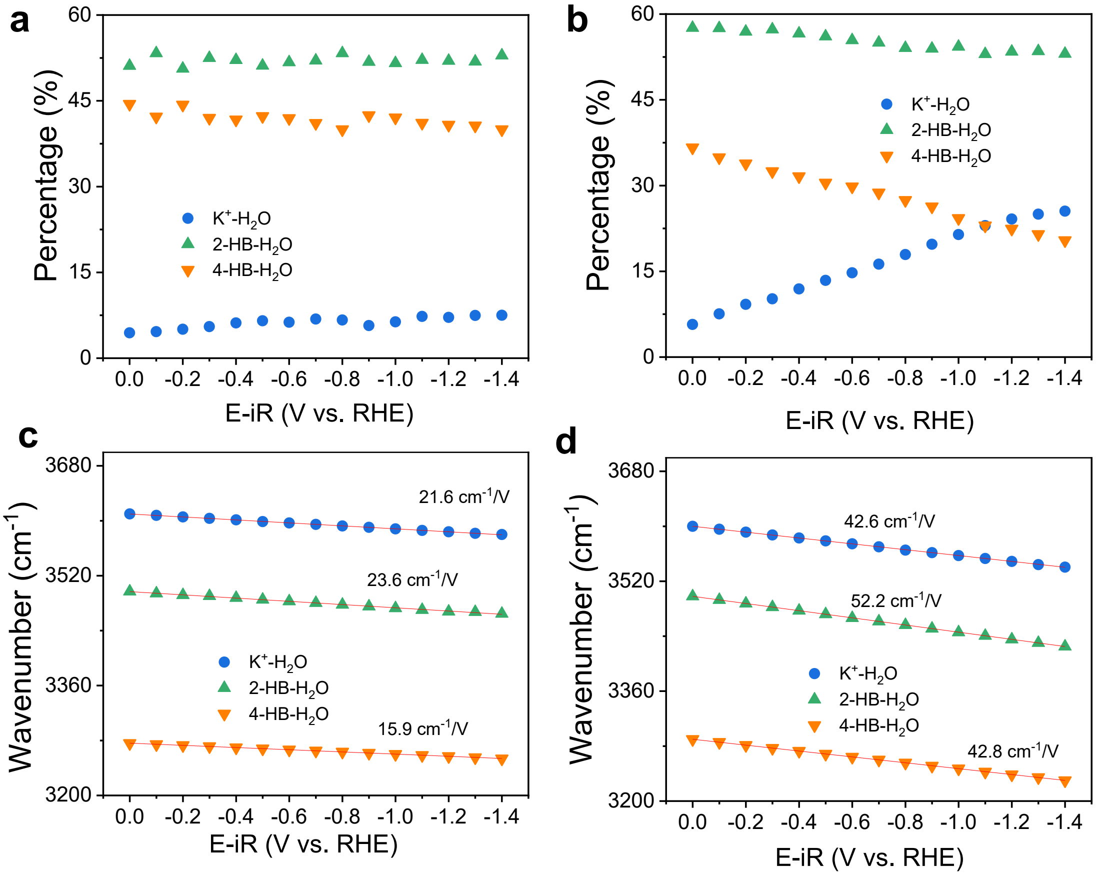

Figure S48. Interfacial water structures. Percentage of different types of interfacial water structures on **a** CuTAPc-layer and **b** CuTAPc. Potential-dependent peak shifts of various interfacial water types in **c** CuTAPc-layer and **d** CuTAPc. Source data for Fig. S48 are provided as a Source Data file.

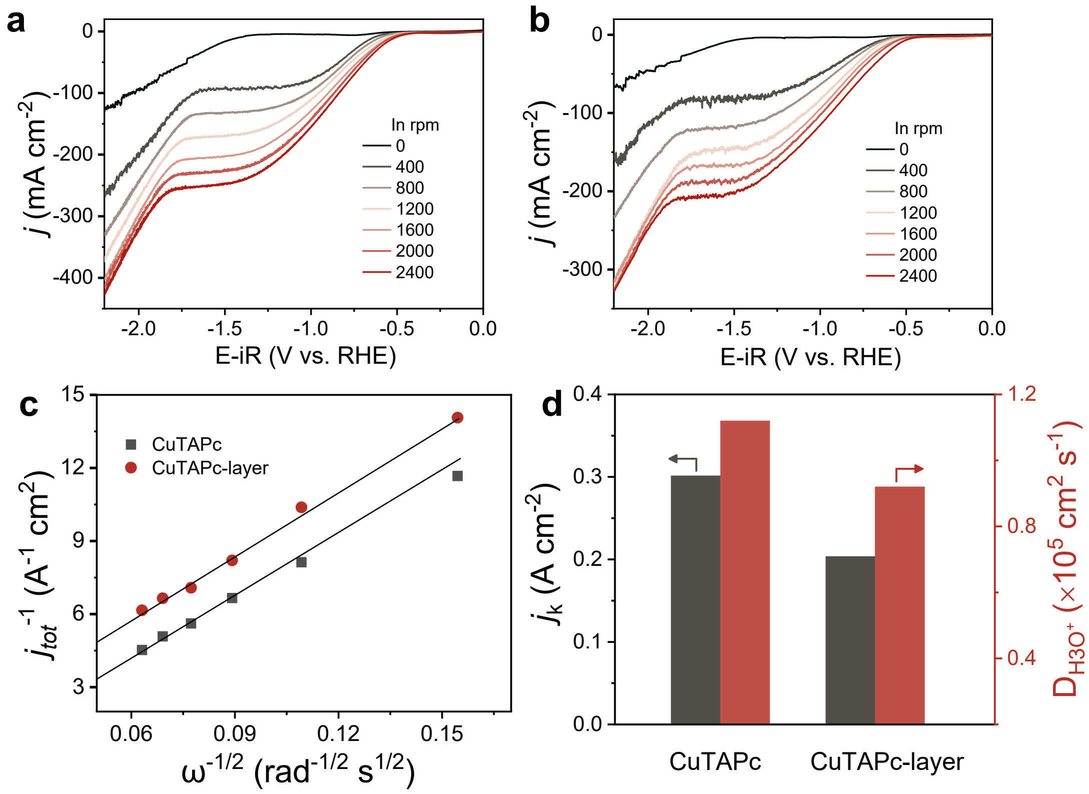

Figure S49. LSV curves for CuTAPc (**a**) and CuTAPc-layer (**b**) in argon-saturated 0.5 M K_2SO_4 + 0.05 M H_2SO_4 at different rotating speeds. **c** Koutecký-Levich plot of different samples at -1.23 V. **d** Kinetic current density of HER and diffusion coefficient of hydronium calculated from Koutecký-Levich equations. Source data for Fig. S49 are provided as a Source Data file.

Supplementary Note 1.

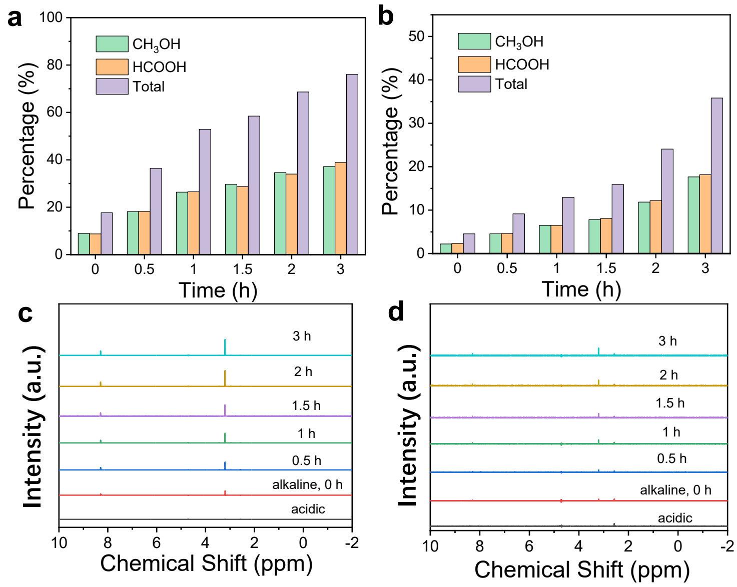

Figure S50. **a**,**c** Disproportionation percentage and corresponding ^{1}H NMR spectra of 1 M FA + 1 M KOH solution at different time intervals. **b**,**d** Disproportionation percentage and corresponding ^{1}H NMR spectra of 0.1 M FA + 1 M KOH solution at different time intervals. Source data for Fig. S50 are provided as a Source Data file.

POM is stable in alkaline conditions, but it readily depolymerizes into FA under acidic conditions. To employ alkaline electrolytes, it is necessary to adjust the solution pH with a strong base. This step inevitably triggers spontaneous FA disproportionation, leading to substantial reactant loss even in the absence of applied potential. As shown in Fig. S50, upon adjusting the solution pH from acidic to alkaline, 17.7% FA is consumed immediately (at 0 h), and 76.1% FA is disproportionated into methanol and formic acid after 3 h. Even at a lower FA concentration (0.1 M FA + 1 M KOH), 35.8% FA is lost after adjusting solution pH for 3 h. These results highlight that alkaline electrolytes will cause significant reactant loss and increase material cost.

Additionally, the need for pH adjustment introduces additional operational costs. KOH is significantly more expensive than other potassium salts such as KCl or K_2SO_4, which accounts for a major cost in electrolysis, as discussed in several recent publications[1,2]. Our approach, which operates under acidic electrolyte conditions, not only eliminates FA losses caused by disproportionation but also avoids the neutralization step and reduces cost arising from the expensive KOH. While our Pt_2Ru alloy for FA oxidation is indeed more expensive than Cu-based alternatives[3,4], its exceptional stability enables extended operational lifetimes. This long-term durability significantly reduces catalyst replacement frequency, resulting in moderate overall catalyst disburse when considered for prolonged operation. Notably, when compared to the membrane, which remains a major contributor to total system cost, the relative expense of the stable Pt_2Ru catalyst becomes less significant, as discussed in some techno-economic analysis[5-10]. Collectively, these factors reinforce the practical and economic advantages of acidic electrolysis for POM conversion.

Supplementary Note 2.

6.3 g of POM solid is first ground and then fully dissolved in 200 mL of 0.5 M H_2SO_4 at 90 °C for 2 h. The resulting POM-derivered FA solution is subsequently subjected to electrocatalytic conversion. The combined CuTAPc-layer/Pt_2Ru catalyst delivers SPCEs of 86.7% for FA-to-CH_3OH and 87.5% for FA-to-HCOOH at a cell voltage of 4 V with a current of 376.3 mA (Fig. S51).

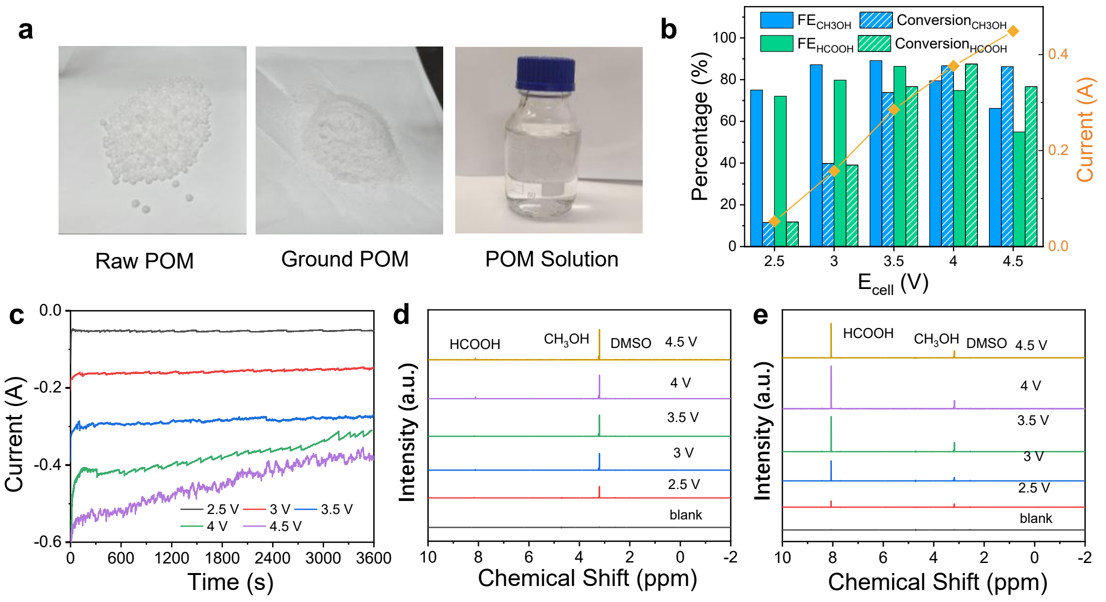

Figure S51. **a** Photograph of raw POM, ground POM, and POM solution. **b** Single pass conversion efficiencies, Faradaic efficiencies, and currents at different cell voltages, catholyte: 1 M FA + 0.5 M K_2SO_4 + 0.05 M H_2SO_4, anolyte: 1 M FA + 0.5 M K_2SO_4 + 0.5 M H_2SO_4. The electrocatalytic performance was measured without iR compensation. **c** Chronoamperometric curves, **d** ^{1}H NMR spectra of catholytes, and **e** ^{1}H NMR spectra of anolytes at different potentials. Source data for Fig. S51 are provided as a Source Data file.

To verify the applicability of the integrated electrocatalyst, practical operating experiments using discarded joint clips and gears as POM substrates are conducted (Fig. S52,53). The POM substrates are mechanically ground and depolymerized in 0.5 M H_2SO_4 at 90 °C for 2 h. CuTAPc-layer/Pt_2Ru system exhibits high performance in waste-derived electrolytes. In joint clips-derived FA electrolytes, it delivers FE_{CH3OH} of 76.3% and FE_{HCOOH} of 70.2% with SPCEs for FA-to-CH_3OH of 81.8% and for FA-to-HCOOH of 80.7% at a cell voltage of 4 V and a current of 369.7 mA (Fig. S52). In gears-derived FA electrolytes, it achieves SPCEs for FA-to-CH_3OH of 82.5% and for FA-to-HCOOH of 80.2% at 4 V and 353.6 mA (Fig. S53). These results demonstrate that the integrated electrocatalyst maintains high selectivity and conversion efficiency with real-world waste POM, supporting its practical viability.

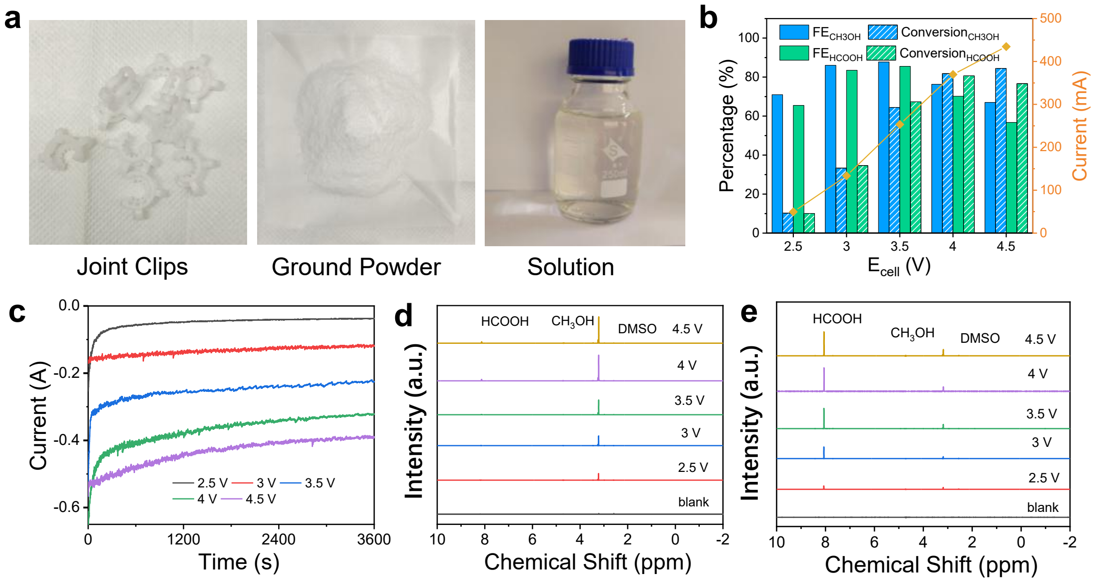

Figure S52. **a** Photograph of waste joint clips, ground joint clips, and joint clips-derived solution. **b** Single pass conversion efficiencies, Faradaic efficiencies, and currents at different cell voltages, catholyte: 1 M FA + 0.5 M K_2SO_4 + 0.05 M H_2SO_4, anolyte: 1 M FA + 0.5 M K_2SO_4 + 0.5 M H_2SO_4. The electrocatalytic performance was measured without iR compensation. **c** Chronoamperometric curves, **d** ^{1}H NMR spectra of catholytes, and **e** ^{1}H NMR spectra of anolytes at different potentials. Source data for Fig. S52 are provided as a Source Data file.

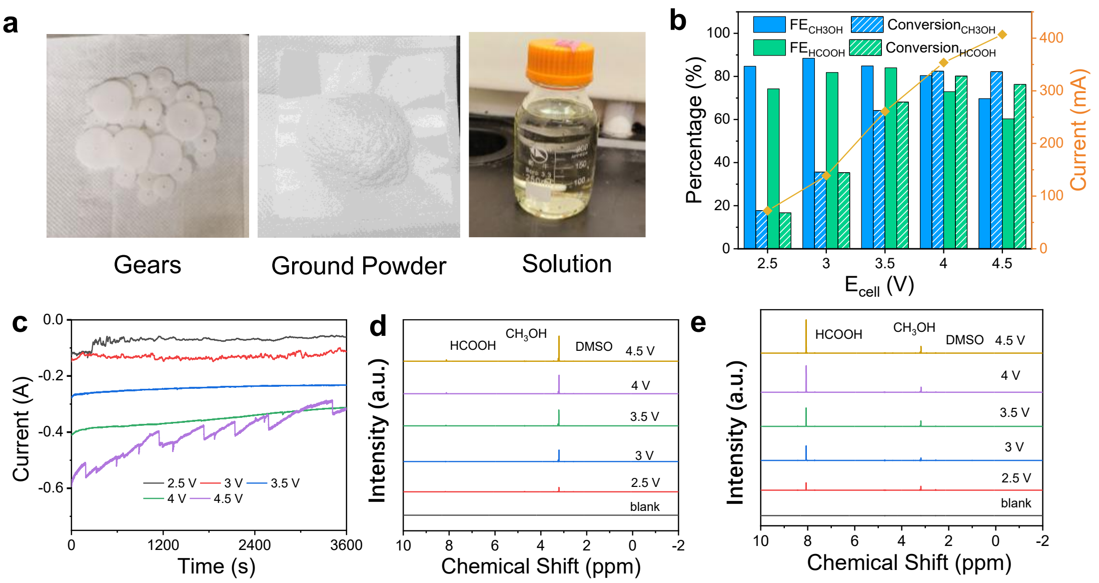

Figure S53. **a** Photograph of waste gears, ground gears, and gears-derived solution. **b** Single pass conversion efficiencies, Faradaic efficiencies, and currents at different cell voltages, catholyte: 1 M FA + 0.5 M K_2SO_4 + 0.05 M H_2SO_4, anolyte: 1 M FA + 0.5 M K_2SO_4 + 0.5 M H_2SO_4. The electrocatalytic performance was measured without iR compensation. **c** Chronoamperometric curves, **d** ^{1}H NMR spectra of catholytes, and **e** ^{1}H NMR spectra of anolytes at different potentials. Source data for Fig. S53 are provided as a Source Data file.

Supplementary Note 3.

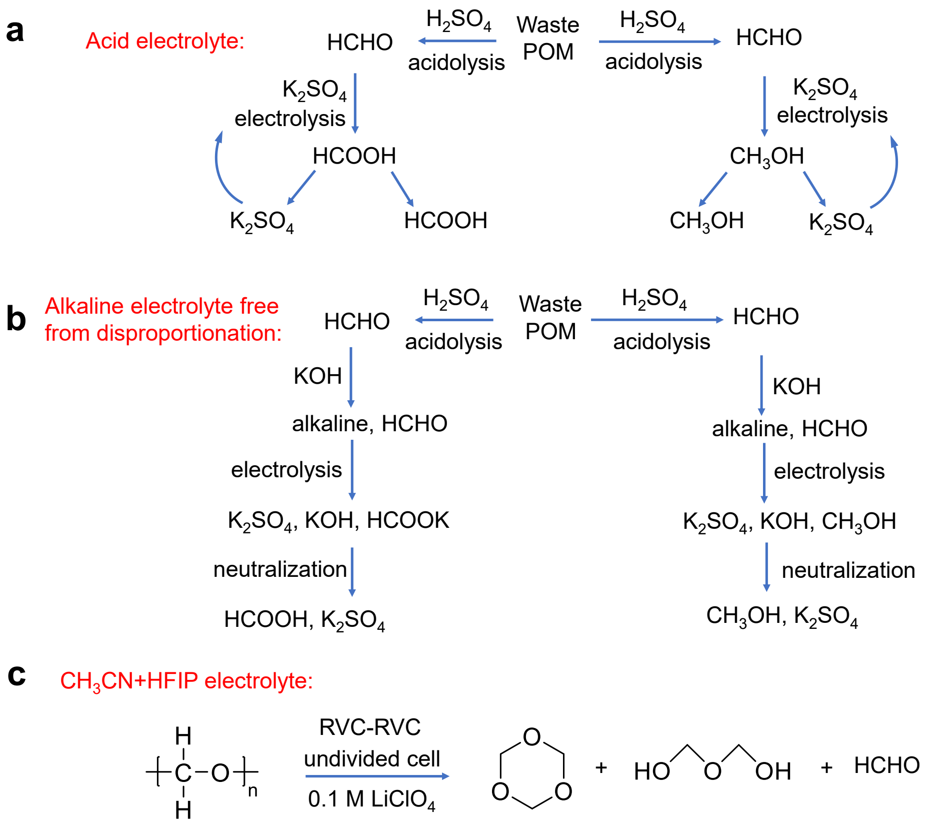

Figure S54. Schematic illustration of POM waste upcycling routes in **a** acidic electrolyte, **b** alkaline electrolyte free from disproportionation, and **c** organic electrolyte.

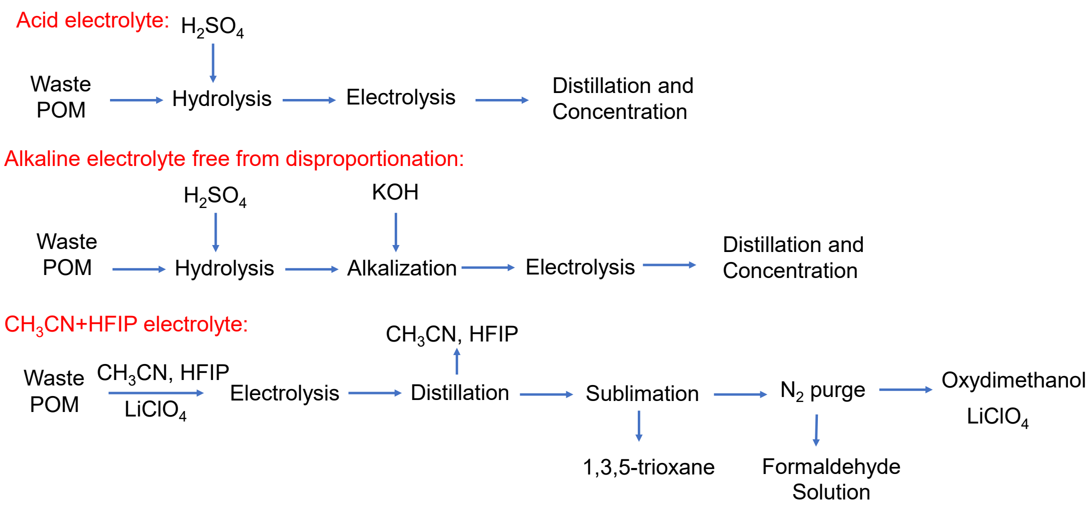

Figure S55. Schematic operating units of the integrated process for POM upcycling in different conditions.

Considering oxydimethanol is extremely unstable and prone to decompose during the subsequent distillation process, assume that oxydimethanol is decomposed to formaldehyde and 1,3,5-trioxane.

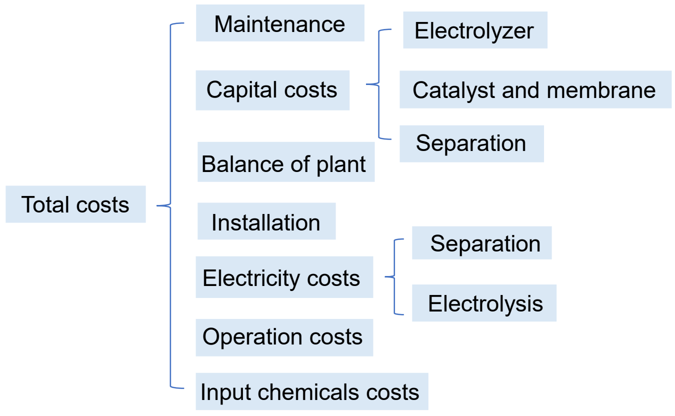

Figure S56. The model used for techno-economic analysis of upcycling waste POM.

We consider six situations of POM depolymerization and upcycling, varying the reaction conditions: acidic electrolysis (Case A); alkaline electrolysis without disproportionation (Case B) and with partial (30%) FA disproportionation (Case C); alkaline conversion with complete disproportionation (Case D); and organic depolymerization, both without (Case E) and with subsequent decomposition of the oxydimethanol product (Case F).

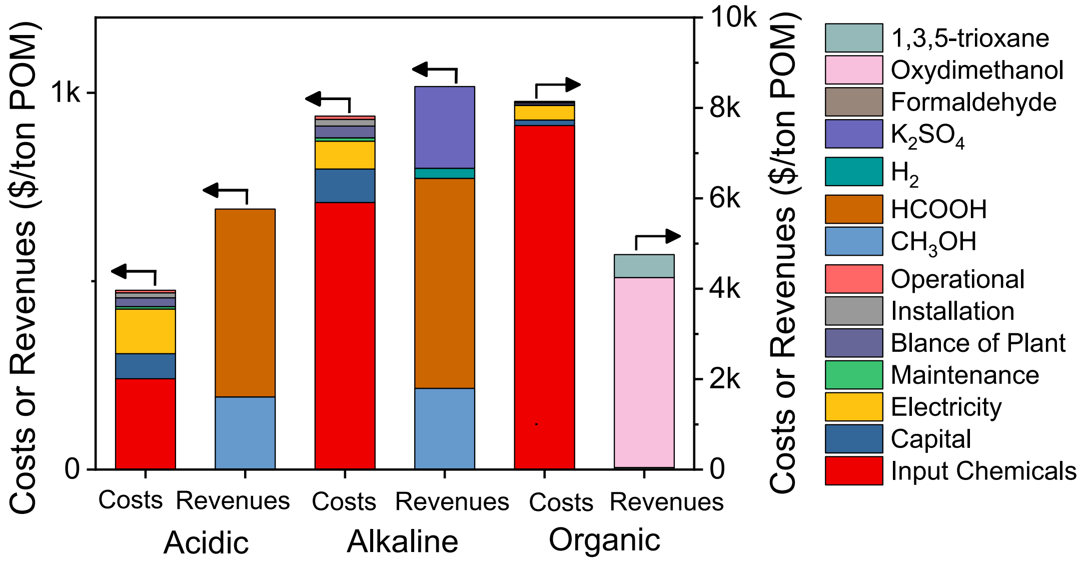

Figure S57. Comparison of the revenues and costs of three electrochemical routes for POM conversion: acidic electrolyte (Case A), alkaline electrolyte without disproportionation (Case B), and organic electrolyte without oxydimethanol decomposition (Case E). Source data for Fig. S57 are provided as a Source Data file.

Listed below are the assumptions made during the calculation process.

1. Assuming that the treatment capacity of the plant is 20 ton of waste POM per day. The waste POM contains ~15% of unreacted impurities.
2. The total cost of the electrolyzer is \$10,000 per m^2.
3. The total catalyst and membrane cost is 5% of the total electrolyzer cost.

4. The capital cost associated with separation equipment is presumed to constitute 10% of electrolyzer capital cost. The separation electricity cost equals 30% of electrolyzer electricity cost.
5. The capacity factor, i.e., the fraction of time the plant is expected to be operational on any given day, is assumed to be 0.8, which means the plant will be operational 19.2 hours a day, and the plant lifetime is assumed to be 20 years.
6. It is assumed that the price of electricity is 3 cents per kilowatt-hour.
7. It is assumed that both operation and maintenance costs amount to 10% of the capital costs.
8. The balance of plant costs is assumed to be 35% of the capital cost. The installation cost is assumed to be 20% of the capital cost.

Table S1. Price of chemicals for calculations[a]

**Product**	**Price ($/ton)**
H_2SO_4	160
POM[b]	600
H_2O	0.22
CH_3OH	500
HCOOH	900
KOH	865
K_2SO_4	300
1,3,5-trioxane	2500
formaldehyde (37%)	200
H_2	1900
1,1,1,3,3,3-hexafluoro-2-propanol	40 $/kg
acetonitrile	1400
lithium perchlorate	20 $/kg
oxydimethanol	10 $/kg

[a]Prices of other chemicals are taken from online trading platforms. (https://www.madein-china.com/)
[b]Assuming that the price of waste POM is 40% of the price of virgin POM.

Case A: upcycling in acid electrolytes

The FEs for CH_3OH and HCOOH in the electrocatalytic FA redox reaction are assumed to be 85%, and the conversion efficiencies of methanol and formic acid from electrolyte are assumed to be 85%.

The calculation processes

1. Capital costs

Based on required cell voltage and current density of 4 V and 93.5 mA cm^{-2}, we can determine the necessary area for the electrolyzer by performing calculations:

$$\text{Area of electrolyzer} = \frac{I}{i}(m^2)$$

Where I is the total consumed electric current; i is the current density (93.5 mA cm^{-2}).

$$I = \frac{Q}{\text{Time in one day} * \text{capacity factor}}$$

Where Q is the total charge.

$$Q = \frac{\text{Mass of methanol} * F * N}{\text{Molar mass of methanol} * \text{Faradaic efficiency}}$$

$$= \frac{0.85 * \frac{0.85}{30} * 10 * 10^6\ g * 96485\ (C/mol) * 2}{0.85} = 5.47 * 10^{10}\ C$$

Where F is the Faraday's constant (96485 C mol^{-1}); N is the electron transfer from FA to methanol

(N=2); Faradaic efficiency is 85%; yield is 85%.

$$I = \frac{Q}{\text{Time in one day} * \text{capacity factor}} = \frac{5.47 * 10^{10}\ \text{C}}{24 * 3600 * 0.8\ \text{s}} = 7.9 * 10^{5}\ \text{A}$$

$$\text{Area of electrolyzer} = \frac{I}{i} = \frac{7.9 * 10^{5}\text{A}}{93.5\ \text{mA cm}^{-2}} = 846\ (\text{m}^2)$$

$$\text{Cost of electrolyzer} = \text{Area} * 10000\ \$\ \text{m}^{-2} = 8.46 * 10^{6}\ \$$$

$$\text{Cost of catalyst and membrane} = \text{Cost of electrolyzer} * 5\% = 4.23 * 10^{5}\ \$$$

$$\text{The capital cost of seperation} = \text{Cost of electrolyzer} \times 10\% = 8.46 * 10^{6} * 10\% = 8.46 * 10^{5}\ \$$$

$$\text{Cost of } K_2SO_4 = \frac{20\ ton * \frac{0.85}{30\frac{g}{mol}} * 2 * 174.26\frac{g}{mol} * 300\ \$/\text{ton}}{2} = 2.96 * 10^{4}\ \$$$

$$\text{Cost of } H_2SO_4 = \frac{20\ ton * \frac{0.85}{30\frac{g}{mol}} * 98.1\frac{g}{mol} * 160\ \$/\text{ton}}{2} = 4.44 * 10^{3}\ \$$$

Therefore, the capital costs component can be calculated as:

$$\text{Capital costs} = \frac{\text{The electrolyzer cost} + \text{The catalyst and membrane costs} + \text{The seperation equipment cost} + K_2SO_4\ \text{cost} + H_2SO_4\ \text{cost}}{\text{Lifetime of plant}}$$

$$= \frac{8.46 * 10^{6} + 4.23 * 10^{5} + 8.46 * 10^{5} + 2.96 * 10^{4} + 4.44 * 10^{3}\ \$}{365 * 20} = 1333.8\ \$\ \text{d}^{-1}$$

2. Electricity costs

The power required to sustain this process can be calculated as follows, assuming an operating cell potential of 4 V:

$$P = \frac{U * I}{1000} = \frac{4\ \text{V} * 7.9 * 10^{5}\ \text{A}}{1000} = 3160\ (\text{KW})$$

$$\text{Electricity consumption per day} = 3160 * 24 * 0.8 = 60672\ (\text{KWhd}^{-1})$$

$$\text{Electricity price} = \text{electricity consumption} * 0.03\ \$\ \text{KWh}^{-1} = 1820.16\ \$\ \text{d}^{-1}$$

$$\text{Electricity cost for separation equipment} = 30\%\ \text{of electrolysis cost} = 546.05\ \$\ \text{d}^{-1}$$

$$\text{Total electricity cost} = \text{electrolysis cost} + \text{seperation cost} = 2366.2\ \$\ \text{d}^{-1}$$

3. Maintenance costs

It is presumed to represent 10% of the capital costs:

$$\text{Maintenance cost per day} = \text{The capital cost} * 10\% = 133.3\ \$\ \text{d}^{-1}$$

4. Balance of plant

$$\text{Balance of plant} = \text{The capital cost} * 35\% = 466.6\ \$\ \text{d}^{-1}$$

5. Installation costs

$$\text{Installation costs} = \text{The capital cost} * 20\% = 266.6\ \$\ \text{d}^{-1}$$

6. Operation costs

$$\text{Operation costs} = \text{The capital cost} * 10\% = 133.3\ \$\ \text{d}^{-1}$$

7. Input chemicals costs

Materials require 20 ton POM, 83 ton H_2O

$$\text{Input chemicals costs} = 20 * 0.4 * 600 + 83 * 0.22 = 4818.3\ \$\ \text{d}^{-1}$$

$$\text{Total costs} = \text{Capital cost} + \text{electricity cost} + \text{maintenance cost} + \text{blance of plant} + \text{installation cost} + \text{operation cost} + \text{input chemical cost} = 1333.8 + 2366.2 + 133.3 + 466.6 + 266.6 + 133.3 + 4818.3\ \$\ d^{-1} = 9488.1\ \$\ d^{-1}$$

8. Output Chemicals

As for the products including CH_3OH, HCOOH

$$\text{The mass of } CH_3OH = 0.85 * 10\ \text{ton} * \frac{0.85}{30\ \text{g/mol}} * 32\ \text{g/mol} = 7.71\ \text{ton}$$

$$\text{The mass of HCOOH} = 0.85 * 10\ \text{ton} * \frac{0.85}{30\ \text{g/mol}} * 46\ \text{g/mol} = 11.08\ \text{ton}$$

$$\text{The total price of out chemicals} = 7.71 * 500 + 11.08 * 900 = 13827\ \$\ d^{-1}$$

$$\text{Profit} = 13827 - 9488.1 = 4338.9\ \$\ d^{-1}$$

Case B: upcycling in alkaline electrolytes, no disproportionation reaction considered

The current density and cell voltage are assumed to be 70 mA cm^{-2} and 2.5 V according to Fig. S41,42. The FEs for CH_3OH and HCOOH in the electrocatalytic FA redox reaction are assumed to be 95%, and the conversion efficiencies of methanol and formate from electrocatalytic FA redox are assumed to be 95%. Assume no disproportionation occurs.

1. Capital cost

$$Q = \frac{\text{Mass of methanol} * F * N}{\text{Molar mass of methanol} * \text{Faradaic efficiency}}$$

$$= \frac{0.85 * 10 * 10^6\ \text{g} * 0.95 * 96485\ \text{C/mol} * 2}{30\ \text{g/mol} * 0.95} = 5.47 * 10^{10}\ \text{C}$$

Where F is the Faraday's constant (96485 C mol^{-1}); N is the electron transfer from FA to methanol (N=2); Faradaic efficiency is 95%; Yield is 95%.

$$I = \frac{Q}{\text{Time in one day} * \text{capacity factor}} = \frac{5.47 * 10^{10}\ \text{C}}{24 * 3600 * 0.8\ \text{s}} = 7.9 * 10^5\ \text{A}$$

$$\text{Area of electrolyzer} = \frac{I}{i} = \frac{7.9 * 10^5 \text{A}}{70\ \text{mA cm}^{-2}} = 1130.5\ (m^2)$$

$$\text{Cost of electrolyzer} = \text{Area} * 10000\ \$\ m^{-2} = 1.13 * 10^7\ \$$$

$$\text{Cost of catalyst and membrane} = \text{Cost of electrolyzer} * 5\% = 5.65 * 10^5\ \$$$

$$\text{The capital cost of seperation equipmen} = \text{Cost of electrolyzer} \times 10\% = 1.13 * 10^7\ \$ * 10\% = 1.13 * 10^6\ \$$$

Therefore, the capital costs component can be calculated as:

$$\text{Capital costs} = \frac{\text{The electrolyzer cost} + \text{The catalyst and membrane costs} + \text{seperation equipment cost}}{\text{Lifetime of plant}}$$

$$= \frac{1.13 * 10^7\ \$ + 5.65 * 10^5 + 1.13 * 10^6\ \$}{365 * 20} = 1780.1\ \$\ d^{-1}$$

2. Electricity costs

The power required to sustain this process can be calculated as follows, assuming an operating cell potential of 2.5 V:

$$P = \frac{U * I}{1000} = \frac{2.5\ \text{V} * 7.9 * 10^5\ \text{A}}{1000} = 1975\ (\text{KW})$$

$$\text{Electricity consumption per day} = 1975 * 24 * 0.8 = 37920\ (\text{KWhd}^{-1})$$

$$\text{Electricity price} = \text{electricity consumption} * 0.03\ \$\ \text{KWh}^{-1} = 1137.6\ \$\ \text{d}^{-1}$$

$$\text{Electricity cost for separation equipment} = 30\%\ \text{of electrolysis cost} = 341.3\ \$\ \text{d}^{-1}$$

$$\text{Total electricity cost} = \text{electrolysis cost} + \text{seperation cost} = 1478.9\ \$\ \text{d}^{-1}$$

3. Maintenance costs

It is presumed to represent 10% of the capital costs:

$$\text{Maintenance cost per day} = \text{The capital cost} * 10\% = 178.01\ \$\ \text{d}^{-1}$$

4. Balance of plant

$$\text{Balance of plant} = \text{The capital cost} * 35\% = 623.04\ \$\ \text{d}^{-1}$$

5. Installation costs

$$\text{Installation costs} = \text{The capital cost} * 20\% = 356.02\ \$\ \text{d}^{-1}$$

6. Operation costs

$$\text{Operation costs} = \text{The capital cost} * 10\% = 178.01\ \$\ \text{d}^{-1}$$

7. Input chemicals costs

Materials require 20 ton POM and 83 ton H_2O. The solution after electrolysis is adjusted to neutral. The final products are HCOOH, CH_3OH, spontaneous-produced H_2, and K_2SO_4 as the products.

$$mass\ (H_2SO_4\ \text{for depolymerization}) = 83 * 10^3\ \text{L} * 0.5\frac{\text{mol}}{\text{L}} * 98.08\frac{\text{g}}{\text{mol}} = 4.07\ \text{ton}$$

$$mass\ (KOH) = 83 * 10^3\ \text{L} * 1\frac{\text{mol}}{\text{L}} * 2 * 56.11\frac{\text{g}}{\text{mol}} = 9.31\ \text{ton}$$

$$mass\ (H_2SO_4\ \text{for neturalization}) = 83 * 10^3\ \text{L} * 0.5\frac{\text{mol}}{\text{L}} * 98.08\frac{\text{g}}{\text{mol}} = 4.07\ \text{ton}$$

$$\text{Input chemicals costs} = 20 * 0.4 * 600 + 9.31 * 865 + 4.07 * 160 * 2 + 83 * 0.22 = 14173.81\ \$\ \text{d}^{-1}$$

$$\begin{aligned}\text{Total costs} &= \text{Capital cost} + \text{electricity cost} + \text{maintenance cost} + \text{blance of plant} \\ &\quad + \text{installation cost} + \text{operation cost} + \text{input chemical cost} \\ &= 1780.1 + 1478.9 + 178.01 + 623.04 + 356.02 + 178.01 + 14173.81\ \$\ \text{d}^{-1} \\ &= 18767.9\ \$\ \text{d}^{-1}\end{aligned}$$

8. Output Chemicals

$$\text{The mass of CH}_3\text{OH} = 0.85 * 10 * \frac{0.95}{30\frac{\text{g}}{\text{mol}}} * 32.04\ \text{g/mol} = 8.62\ \text{ton}$$

$$\text{The mass of HCOOH} = 0.85 * 10 * \frac{0.95}{30\frac{\text{g}}{\text{mol}}} * 46.05\ \text{g/mol} = 12.39\ \text{ton}$$

$$\text{The mass of K}_2\text{SO}_4 = 83 * 10^3\ \text{L} * 0.5\frac{\text{mol}}{\text{L}} * 2 * 174.26\ \text{g/mol} = 14.46\ \text{ton}$$

$$\text{The mass of H}_2 = 10 * \frac{\frac{0.85}{30}}{2} * 2\ \text{g/mol} = 0.28\ \text{ton}$$

$$\begin{aligned}\text{The total price of out chemicals} &= 8.62 * 500 + 12.39 * 900 + 14.46 * 300 + 0.28 * 1900 \\ &= 20331\ \$\ \text{d}^{-1}\end{aligned}$$

$$\text{Profit} = 20331 - 18767.9 = 1563.1\ \$\ \text{d}^{-1}$$

Case C: upcycling in alkaline electrolytes, 30% of POM-derived FA is disproportionated

The current density and cell voltage are assumed to be those in alkaline electrolytes without

disproportionation reaction. The FEs for CH_3OH and HCOOH in the electrocatalytic FA redox reaction are assumed to be 70%, and the conversion efficiencies of methanol and formate from electrocatalytic FA redox are assumed to be 70%. The separation costs are assumed to be 3 timies of case B.

1. Capital cost

$$Q = \frac{\text{Mass of methanol} * F * N}{\text{Molar mass of methanol} * \text{Faradaic efficiency}}$$

$$= \frac{0.85 * 10 * 10^6 \text{ g} * 0.7 * 96485 \text{ C/mol} * 2}{30 \text{ g/mol} * 0.7} = 5.47 * 10^{10} \text{ C}$$

Where F is the Faraday's constant (96485 C mol^{-1}); N is the electron transfer from FA to methanol (N=2); Faradaic efficiency is 95%; Yield is 95%.

$$I = \frac{Q}{\text{Time in one day} * \text{capacity factor}} = \frac{5.47 * 10^{10} \text{ C}}{24 * 3600 * 0.8 \text{ s}} = 7.9 * 10^5 \text{ A}$$

$$\text{Area of electrolyzer} = \frac{I}{i} = \frac{7.9 * 10^5 \text{A}}{70 \text{ mA cm}^{-2}} = 1130.5 \ (\text{m}^2)$$

$$\text{Cost of electrolyzer} = \text{Area} * 10000 \ \$ \ \text{m}^{-2} = 1.13 * 10^7 \ \$$$

$$\text{Cost of catalyst and membrane} = \text{Cost of electrolyzer} * 5\% = 5.65 * 10^5 \ \$$$

$$\text{The capital cost of seperation equipment} = \text{Cost of electrolyzer} \times 30\%$$
$$= 1.13 * 10^7 \ \$ * 30\% = 3.39 * 10^6 \ \$$$

Therefore, the capital costs component can be calculated as:

$$\text{Capital costs} = \frac{\text{The electrolyzer cost} + \text{The catalyst and membrane costs} + \text{seperation equipment cost}}{\text{Lifetime of plant}}$$

$$= \frac{1.13 * 10^7 \ \$ + 5.65 * 10^5 + 3.39 * 10^6 \ \$}{365 * 20} = 2089.72 \ \$ \ \text{d}^{-1}$$

2. Electricity costs

The power required to sustain this process can be calculated as follows, assuming an operating cell potential of 2 V:

$$P = \frac{U * I}{1000} = \frac{2 \text{ V} * 7.9 * 10^5 \text{ A}}{1000} = 1580 \ (\text{KW})$$

$$\text{Electricity consumption per day} = 1580 * 24 * 0.8 = 30336 \ (\text{KWhd}^{-1})$$

$$\text{Electricity price} = \text{electricity consumption} * 0.03 \ \$ \ \text{KWh}^{-1} = 910.08 \ \$ \ \text{d}^{-1}$$

$$\text{Electricity cost for separation equipment} = 90\% \text{ of electrolysis cost} = 819.07 \ \$ \ \text{d}^{-1}$$

$$\text{Total electricity cost} = \text{electrolysis cost} + \text{seperation cost} = 1729.15 \ \$ \ \text{d}^{-1}$$

3. Maintenance costs

It is presumed to represent 10% of the capital costs:

$$\text{Maintenance cost per day} = \text{The capital cost} * 10\% = 208.97 \ \$ \ \text{d}^{-1}$$

4. Balance of plant

$$\text{Balance of plant} = \text{The capital cost} * 35\% = 731.4 \ \$ \ \text{d}^{-1}$$

5. Installation costs

$$\text{Installation costs} = \text{The capital cost} * 20\% = 417.94 \ \$ \ \text{d}^{-1}$$

6. Operation costs

$$\text{Operation costs} = \text{The capital cost} * 10\% = 208.97 \ \$ \ \text{d}^{-1}$$

7. Input chemicals costs

Materials require 20 ton POM and 83 ton H_2O. The solution after electrolysis is adjusted to neutral. The final products are HCOOH, CH_3OH, spontaneous-produced H_2, and K_2SO_4 as the products.

$$mass\ (H_2SO_4 \text{ for depolymerization}) = 83 * 10^3 \text{ L} * 0.5\frac{\text{mol}}{\text{L}} * 98.08\frac{\text{g}}{\text{mol}} = 4.07 \text{ ton}$$

$$mass\ (KOH) = 83 * 10^3 \text{ L} * 1\frac{\text{mol}}{\text{L}} * 2 * 56.11\frac{\text{g}}{\text{mol}} = 9.31 \text{ ton}$$

$$mass\ (H_2SO_4 \text{ for neturalization}) = 83 * 10^3 \text{ L} * 0.5\frac{\text{mol}}{\text{L}} * 98.08\frac{\text{g}}{\text{mol}} = 4.07 \text{ ton}$$

$$\text{Input chemicals costs} = 20 * 0.4 * 600 + 9.31 * 865 + 4.07 * 160 * 2 + 83 * 0.22 = 14173.81 \text{ \$ d}^{-1}$$

$$\begin{aligned}\text{Total costs} &= \text{Capital cost} + \text{electricity cost} + \text{maintenance cost} + \text{blance of plant} \\ &+ \text{installation cost} + \text{operation cost} + \text{input chemical cost} \\ &= 2089.72 + 1729.15 + 208.97 + 731.4 + 417.94 + 208.97 \\ &+ 14173.81 \text{ \$ d}^{-1} = 19559.94 \text{ \$ d}^{-1}\end{aligned}$$

8. Output Chemicals

$$\text{The mass of } CH_3OH_{\text{disproportionation}} = 0.85 * 10 * \frac{0.3}{30\frac{\text{g}}{\text{mol}}} * 32.04 \text{ g/mol} = 2.72 \text{ ton}$$

$$\text{The mass of } CH_3OH_{\text{reduction}} = 0.7 * 0.85 * 10 * \frac{0.7}{30\frac{\text{g}}{\text{mol}}} * 32.04 \text{ g/mol} = 4.45 \text{ ton}$$

$$\text{The mass of } HCOOH_{\text{disproportionation}} = 0.85 * 10 * \frac{0.3}{30\frac{\text{g}}{\text{mol}}} * 46.05 \text{ g/mol} = 3.91 \text{ ton}$$

$$\text{The mass of } HCOOH_{\text{oxidation}} = 0.7 * 0.85 * 10 * \frac{0.7}{30\frac{\text{g}}{\text{mol}}} * 46.05 \text{ g/mol} = 6.39 \text{ ton}$$

$$\text{The mass of } K_2SO_4 = 83 * 10^3 \text{ L} * 0.5\frac{\text{mol}}{\text{L}} * 2 * 174.26 \text{ g/mol} = 14.46 \text{ ton}$$

$$\text{The mass of } H_2 = 0.7 * 0.7 * 10 * \frac{\frac{0.85}{30}}{2} * 2 \text{ g/mol} = 0.14 \text{ ton}$$

$$\begin{aligned}&\text{The total price of out chemicals} \\ &= (2.72 + 4.45) * 500 + (3.91 + 6.39) * 900 + 14.46 * 300 + 0.14 * 1900 \\ &= 17459 \text{ \$ d}^{-1}\end{aligned}$$

$$\text{Profit} = 17459 - 19559.94 = -2100.9 \text{ \$ d}^{-1}$$

Case D: upcycling in alkaline electrolytes, only disproportionation occurs

We assume that the extent of FA for disproportionation reaction is 80%. The presence of FA in obtained solution will increase the separation cost, and is 3 times that of Case C.

1. Separation cost

$$\text{The capital cost of seperation equipment} = \text{Case C} \times 3 = 3 * 3.39 * 10^6 = 10.2 * 10^6 \text{ \$}$$

$$\text{Electricity cost for separation equipment} = \text{Case C} \times 3 = 2457.2 \text{ \$ d}^{-1}$$

$$\text{Sepapration costs} = \frac{\text{seperation equipment cost}}{\text{Lifetime of plant}} + \text{ separation electricity}$$

$$= \frac{10.2 * 10^6}{20 * 365} + 2457.2 = 1397.3 + 2457.2 = 3854.5 \ \$ \ \text{d}^{-1}$$

2. Maintenance costs

It is presumed to represent 10% of the capital costs:

$$\text{Maintenance cost per day} = \text{The capital cost} * 10\% = 139.7 \ \$ \ \text{d}^{-1}$$

3. Balance of plant

$$\text{Balance of plant} = \text{The capital cost} * 35\% = 489 \ \$ \ \text{d}^{-1}$$

4. Installation costs

$$\text{Installation costs} = \text{The capital cost} * 20\% = 279.5 \ \$ \ \text{d}^{-1}$$

5. Operation costs

$$\text{Operation costs} = \text{The capital cost} * 10\% = 139.7 \ \$ \ \text{d}^{-1}$$

6. Input Chemicals

Materials require 20 ton POM and 83 ton H_2O. The solution after electrolysis is adjusted to neutral. The final products are HCOOH, CH_3OH, and K_2SO_4 as the products.

$$mass\ (H_2SO_4 \text{ for depolymerization}) = 83 * 10^3 \text{ L} * 0.5 \frac{\text{mol}}{\text{L}} * 98.08 \frac{\text{g}}{\text{mol}} = 4.07 \text{ ton}$$

$$mass\ (KOH) = 83 * 10^3 \text{ L} * 1 \frac{\text{mol}}{\text{L}} * 2 * 56.11 \frac{\text{g}}{\text{mol}} = 9.31 \text{ ton}$$

$$mass\ (H_2SO_4 \text{ for neturalization}) = 83 * 10^3 \text{ L} * 0.5 \frac{\text{mol}}{\text{L}} * 98.08 \frac{\text{g}}{\text{mol}} = 4.07 \text{ ton}$$

$\text{Input chemicals costs} = 20 * 0.4 * 600 + 9.31 * 865 + 4.07 * 160 * 2 + 83 * 0.22 = 14173.8 \ \$ \ \text{d}^{-1}$

$$\text{Total costs} = 3854.5 + 139.7 + 489 + 279.5 + 139.7 + 14173.8 = 19076.2 \ \$ \ \text{d}^{-1}$$

7. Output Chemicals

$$\text{The mass of } CH_3OH = 0.85 * 10 * \frac{0.8}{30 \frac{\text{g}}{\text{mol}}} * 32.04 \frac{\text{g}}{\text{mol}} = 7.3 \text{ ton}$$

$$\text{The mass of HCOOH} = 0.85 * 10 * \frac{0.8}{30 \frac{\text{g}}{\text{mol}}} * 46.05 \frac{\text{g}}{\text{mol}} = 10.4 \text{ ton}$$

$$\text{The mass of } K_2SO_4 = 83 * 10^3 \text{ L} * 0.5 \frac{\text{mol}}{\text{L}} * 2 * 174.26 \text{ g/mol} = 14.46 \text{ ton}$$

$$\text{The total price of out chemicals} = 7.3 * 500 + 10.4 * 900 + 14.46 * 300 = 17348 \ \$ \ \text{d}^{-1}$$

$$\text{Profit} = 17348 - 19076.2 = -1728.2 \ \$ \ \text{d}^{-1}$$

Case E: electromediated depolymerization of POM, data from *Ref. 12*:

In this work[12], 105 mg of POM is depolymerized at 60 °C in an undivided cell containing 26 mL of CH_3CN, 4 mL of HFIP, and 0.1 M $LiClO_4$ as the electrolyte. The product yields of formaldehyde, oxydimethanol, and 1,3,5-trioxane are 10%, 19%, and 8%, respectively. The depolymerization of POM is driven by protons generated from the electrochemical oxidation of HFIP. We assume that about no HFIP is converted to 1,1,1,3,3,3-hexafluoro-2-propanone. The reaction is carried out at applied potential of 3.5 V vs. Ag/AgCl; we assume a cell voltage of 7 V and the current is calculated:

$$\mathrm{n(POM)} = \frac{105\ \mathrm{mg}}{30\,\frac{\mathrm{g}}{\mathrm{mol}}} = 3.5\ \mathrm{mmol}$$

1,1,1,3,3,3-hexafluoro-2-propanol (HFIP) undergoes electrooxidation at the anode to form 1,1,1,3,3,3-hexafluoropropan-2-one with the transfer of 2 electrons. The produced protons are utilized to depolymerize the POM chain. The amount of POM is assumed to be equivalent to the amount of reacted HFIP.

$$\mathrm{Q = n * N * F = 2 * 3.5\ mmol * 96485\,\frac{C}{mol} = 675.4\ C = i * t}$$

$$\mathrm{Currrent} = \frac{675.4\ \mathrm{C}}{2 * 1800\ \mathrm{s}} = 187.6\ \mathrm{mA}$$

The area of reticulated vitreous carbon (RVC) foam is 1.8 cm^2.

The current density:

$$\mathrm{i} = \frac{187.6\ \mathrm{mA}}{1.8\ cm^2} = 104.2\ \mathrm{mA}\ cm^{-2}$$

Assuming a treatment capacity of the plant is 20 ton of waste POM per day.

1. Capital cost

$$\mathrm{n(POM) = n(HFIP)} = 0.85 * 20 * \frac{10^6}{30} = 5.67 * 10^5\ mol$$

$$\mathrm{Q = n(HFIP)} * F * N = 5.67 * 10^5 * 2 * 96485 = 1.09 * 10^{11}\ C$$

Where F is the Faraday's constant (96485 C mol^{-1}); N is the electron transfer from HFIP to 1,1,1,3,3,3-hexafluoropropan-2-one (N=2).

$$\mathrm{I} = \frac{\mathrm{Q}}{\mathrm{Time\ in\ one\ day * capacity\ factor}} = \frac{1.09 * 10^{11}\ \mathrm{C}}{24 * 3600 * 0.8\ \mathrm{s}} = 1.58 * 10^6\ \mathrm{A}$$

$$\mathrm{Area\ of\ electrolyzer} = \frac{\mathrm{I}}{\mathrm{i}} = \frac{1.58 * 10^6\ \mathrm{A}}{104.2\ \mathrm{mA\ cm^{-2}}} = 1518.26\ (\mathrm{m^2})$$

$$\mathrm{Cost\ of\ electrolyzer = Area * 10000\ \$\ m^{-2} = 1.52 * 10^7\ \$}$$

$$\mathrm{Cost\ of\ catalyst\ and\ membrane = Cost\ of\ electrolyzer * 5\% = 7.6 * 10^5\ \$}$$

$$\mathrm{The\ capital\ cost\ of\ seperation\ equipment\ = Cost\ of\ electrolyzer \times\ 10\%\ = 1.54 * 10^6\ \$\ d^{-1}}$$

Therefore, the capital costs component can be calculated as:

$$\mathrm{Capital\ costs} = \frac{\mathrm{The\ electrolyzer\ cost\ +\ The\ catalyst\ and\ membrane\ costs + seperation\ equipment\ cost}}{\mathrm{Lifetime\ of\ plant}}$$

$$= \frac{1.54 * 10^7\ \$\ + 7.6 * 10^5\ \$ + 1.54 * 10^6}{365 * 20} = 2424.66\ \$\ \mathrm{d^{-1}}$$

2. Electricity costs

The power required to sustain this process can be calculated as follows, assuming an operating cell potential of 7 V:

$$\mathrm{P} = \frac{\mathrm{U * I}}{1000} = \frac{7\ \mathrm{V} * 1.6 * 10^6\ \mathrm{A}}{1000} = 11200\ (\mathrm{KW})$$

$$\mathrm{Electricity\ consumption\ per\ day = 11200 * 24 * 0.8 = 215040\ (KWhd^{-1})}$$

$$\mathrm{Electricity\ price = electricity\ consumption * 0.03\ \$\ KWh^{-1} =\ 6451.26\ \$\ d^{-1}}$$

3. Maintenance costs

It is presumed to represent 10% of the capital costs

$$\mathrm{Maintenance\ cost\ per\ day\ =\ The\ capital\ cost *\ 10\% = 242.46\ \$\ d^{-1}}$$

4. Balance of plant

$$\text{Balance of plant} = \text{The capital cost} * 35\% = 848.63\ \$\ \mathrm{d}^{-1}$$

5. Installation costs

$$\text{Installation costs} = \text{The capital cost} * 20\% = 484.93\ \$\ \mathrm{d}^{-1}$$

6. Operation costs

$$\text{Operation costs} = \text{The capital cost} * 10\% = 242.47\ \$\ \mathrm{d}^{-1}$$

7. Input chemicals costs

Materials require 20 ton POM.

$$\text{The chemical cost of HFIP} = 20\ ton * 0.85 * \frac{4\ mL}{105\ mg} * 1.6\frac{g}{mL} * 40\ \$/\text{kg} = 4.14 * 10^{7}\ \$$$

$$\text{The chemical cost of } \mathrm{CH_3CN} = 20\ ton * 0.85 * \frac{26\ mL}{105\ mg} * 0.79\frac{g}{mL} * 1400\ \$/\text{ton}$$

$$= 4.66 * 10^{6}\ \$$$

$$\text{The chemical cost of } \mathrm{LiClO_4} = 20\ ton * 0.85 * \frac{30\ mL * 0.1\frac{mol}{mL}}{105\ mg} * 106.4\frac{g}{mol} * 20\ \$/\text{kg}$$

$$= 1.03 * 10^{9}\ \$$$

$$\text{Input chemicals costs} = \text{POM cost} + \frac{\text{HFIP cost} + \mathrm{CH_3CN}\text{ cost} + \mathrm{LiClO_4}\text{cost}}{\text{Lifetime of plant}} = 20 * 0.4 * 600 +$$

$$\frac{4.14*10^{7}+4.66*10^{6}+1.03*10^{9}}{365*20} = 152205\ \$\ \mathrm{d}^{-1}$$

$$\text{Total costs} = \text{Capital cost} + \text{electricity cost} + \text{maintenance cost} + \text{blance of plant} + \text{installation cost} + \text{operation cost} + \text{input chemical cost}$$

$$= 149823 + 6451.26 + 239.73 + 839 + 479.45 + 239.73 + 4800\ \$\ \mathrm{d}^{-1}$$

$$= 162872.2\ \$\ \mathrm{d}^{-1}$$

8. Output Chemicals

$$\text{The mass of formaldehyde} = 0.85 * 20\ \text{ton} * 10\% = 1.7\ \text{ton}$$

$$\text{The mass of oxydimethanol} = 0.85 * \frac{20\ \text{ton}}{30\frac{g}{mol}} * 19\% * 78\ g/mol = 8.4\ \text{ton}$$

$$\text{The mass of 1,3,5} - \text{trioxane} = 0.85 * \frac{20\ \text{ton}}{30\frac{g}{mol}} * 8\% * 90\ g/mol = 4.08\ \text{ton}$$

$$\text{The total price of } out\ chemicals = 1.7 * \frac{200}{0.37} + 8.4 * 10 * 1000 + 2500 * 4.08$$

$$= 95118.9\ \$\ \mathrm{d}^{-1}$$

$$\text{Profit} = 95118.9 - 162872.2 = -67753.3\ \$\ \mathrm{d}^{-1}$$

Case F. Oxydimethanol is unstable and decomposed.

We assume the cost for case F is the same as case E. Given that oxydimethanol is extremely unstable and tends to decompose during the subsequent distillation, we assume it breaks down into formaldehyde and 1,3,5-trioxane in a 1:1 atomic ratio. Therefore,

$$\text{The mass of formaldehyde} = 0.85 * 20\ \text{ton} * 10\% + 0.85 * 20\ ton * 9.5\% = 3.32\text{ton}$$

$$\text{The mass of } 1,3,5-\text{trioxane}$$

$$= 0.85 * \frac{20 \text{ ton}}{30\frac{g}{mol}} * 8\% * 90\frac{g}{mol} + 0.85 * \frac{20 \text{ ton}}{30\frac{g}{mol}} * 19\% * 0.5 * \frac{1}{3} * 90\frac{g}{mol}$$

$$= 5.7 \text{ ton}$$

$$\text{The total price of } \textit{out chemicals} = 3.32 * \frac{200}{0.37} + 2500 * 5.7 = 16044.6 \ \$ \ \mathrm{d}^{-1}$$

$$\text{Profit} = 16044.6 - 162872.2 = -146827.6 \ \$ \ \mathrm{d}^{-1}$$

Table S1. Operating costs and revenues of electrochemical upcycling POM waste in various media.

Case	A	B	C	D	E	F
Costs						
Capital	1333.8	1780.1	2089.7	1397.3	2424.7	2424.7
Electricity	2366.2	1478.9	910.1	2457.2	6451.3	6451.3
Maintenance	133.3	178	209	139.7	242.5	242.5
Balance of plant	466.6	623	731.4	489	848.6	848.6
Installation	266.6	356	417.9	279.5	484.9	484.9
Operation	133.3	178	209	139.7	242.5	242.5
Input chemicals	4818.3	14173.8	14173.81	14173.8	152205	152205
Total Costs	9488.1	18767.9	19559.94	19076.2	162899.5	162899.5
Output chemicals						
CH_3OH	3855	4310	3585	3650	/	/
HCOOH	9972	11151	9270	9360	/	/
H_2	/	532	266	/	/	/
K_2SO_4	/	4338	4338	4338	/	/
formaldehyde	/	/	/	/	918.9	1794.6
1,3,5-trioxane	/	/	/	/	10200	14250
oxydimethanol	/	/	/	/	84000	/
Revenues	13827	20331	17459	17348	95118.9	16044.6
Profit						
Profit	4388.9	1563.1	-2100.9	-1728.2	-67780.6	-146854.9

[a]Assuming that the treatment capacity of the plant is 20 ton of waste POM per day.

[b]Case A refers to acidic electrolysis.

[c]Case B refers to alkaline electrolysis free from disproportionation.

[d]Case C refers to alkaline electrolysis, 30% FA occurs disproportionation.

[e]Case D refers to alkaline disproportionation.

[f]Case E refers to organic depolymerization.

[g]Case F refers to organic depolymerization, oxydimethanol decomposes.

Supplementary Note 4.

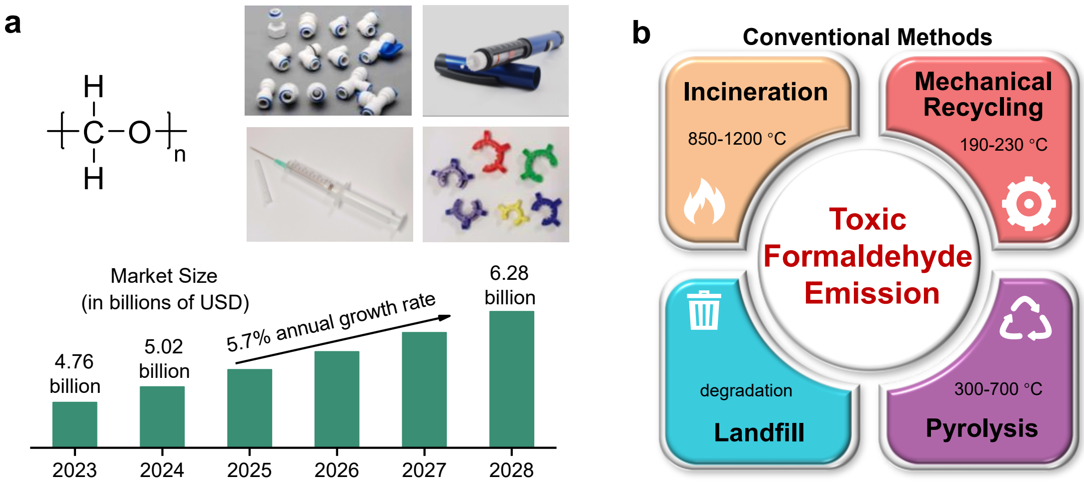

Figure S58. **a** Structure, application, and projected market size of polyoxymethylene. **b** Conventional methods for degrading POM waste. Source data for Fig. S58 are provided as a Source Data file.

Supplementary Note 5.

Tabel S2. Uncompensated solution resistance of various catalysts measured in the H-cell.

	CuTAPc	CuTAPc-layer	CoTAPc	CoTAPc-layer	Pt_2Ru	20% Pd/C	Ru	Cu	40% Au/C	Ag	Pt	Cu_2O
Resistance (Ω)	2.1	2.2	2.2	2.1	2	2	2.2	2.3	2.1	2.1	2.3	2.5

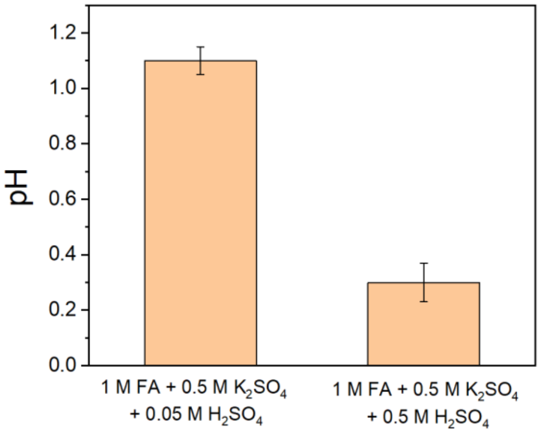

Figure S59. Measured pH values for the two electrolyte solutions:1 M FA + 0.5 M K_2SO_4 + 0.05 M H_2SO_4 and 1 M FA + 0.5 M K_2SO_4 + 0.5 M H_2SO_4. Source data for Fig. S59 are provided as a Source Data file.

Reference:

1. Erick Huang, J. et al. CO_2 electrolysis to multicarbon products in strong acid. *Science* **372**, 1074-1078 (2021).
2. Ren, Y. *et al.* Selective electrooxidation of 5-hydroxymethylfurfural at pilot scale by engineering a solid polymer electrolyte reactor. *Nat. Catal.* **8**, 771-783 (2025).

3. Gao, X. et al. Enhancing the stability of Cu-based electrocatalyst via Fe alloy in electrocatalytic formaldehyde oxidation with long durability. *Adv. Funct. Mater.* **35**, 2417545 (2025).
4. Yang, M. et al. A self-reactivated PdCu catalyst for aldehyde electro-oxidation with anodic hydrogen production. *Nat. Commun.* **15**, 9852 (2024).
5. Ding, W. *et al.* Rapid surface reconstruction of amorphous-crystalline NiO for industrial-scale electrocatalytic PET upcycling. *Angew. Chem. Int. Ed.* **64**, e202418640 (2025).
6. Chen, Q. *et al.* Catalytic oxidation upcycling of polyethylene terephthalate to commodity carboxylic acids. *Nat. Commun.* ***15,*** 10732 (2024).
7. Leow, W. R. *et al.* Chloride-Mediated Selective Electrosynthesis of Ethylene and Propylene Oxides at High Current Density. *Science* **368**, 1228-1233 (2020).
8. Zhou, H. *et al.* Electrocatalytic upcycling of polyethylene terephthalate to commodity chemicals and H_2 fuel. *Nat. Commun.* **12**, 4679 (2021).
9. Zhao, B. H. *et al.* Economically viable electrocatalytic ethylene production with high yield and selectivity. *Nat. Sustain.* **6**, 827-837 (2023).
10. Shi, Q. *et al.* Electrocatalytic upgrading of plastic and biomass-derived polyols to formamide under ambient conditions. *Angew. Chem. Int. Ed.* **63**, e202407580 (2024).
11. Li, Y., Wei, X., Han, S., Chen, L. & Shi, J. MnO_2 electrocatalysts coordinating alcohol oxidation for ultra-durable hydrogen and chemical productions in acidic solutions. *Angew. Chem. Int. Ed.* **60**, 21464-21472 (2021).
12. Zhou, Y., Rodríguez-López, J. & Moore, J.S. Heterogenous electromediated depolymerization of highly crystalline polyoxymethylene. *Nat. Commun.* **14**, 4847 (2023).
