# Supplementary material for: Electrochemically mediated disproportionation for selective formaldehyde upcycling in acid
Source: Nat Commun. 2026 Mar 18;17:4120. doi: 10.1038/s41467-026-70739-y (PMC13150010; doi:10.1038/s41467-026-70739-y)
Supplement: Supplementary file 2 — Description of Additional Supplementary Files [file 41467_2026_70739_MOESM2_ESM.pdf]

Description of Additional Supplementary Files

File Name: Supplementary Data 1

Description: Structure of *H_2CO.

File Name: Supplementary Data 2

Description: Structure of *$OHCH_2OH$.

File Name: Supplementary Data 3

Description: Structure of *$OHCH_2O$.

File Name: Supplementary Data 4

Description: Structure of *OHCHO.
